# Flow Characteristics of Unclassified Tailings Backfill Slurry and Optimization of Roof-Contact Backfilling Scheme

**DOI:** 10.3390/ma19173580

**Published:** 2026-08-24

**Authors:** Hongjiao Li, Yuye Tan, Xu Huang, Zenggui Zhang, Jiazhao Chen, Yuchao Deng

**Affiliations:** 1 Key Laboratory of the Ministry of Education for High-Efficiency Mining and Safety in Metal Mines, University of Science and Technology Beijing, Beijing 100083, China; m202520091@xs.ustb.edu.cn (H.L.); huangx2000@126.com (X.H.); cjzgzyx2021@163.com (J.C.); d1938545696@163.com (Y.D.); 2 School of Resources and Safety Engineering, University of Science and Technology Beijing, Beijing 100083, China; 3 Nanchang Branch, Beijing Sloco Resource Technology Co., Ltd., Nanchang 330006, China; 4 Daye Iron Mine Co., Ltd., WISCO Resources Group, Wuhan Iron and Steel Group Co., Ltd., Huangshi 435006, China; e85381@baostel.com

**Keywords:** unclassified tailings backfill slurry, flow characteristics, rheological parameters, inversion model, roof-contact backfilling

## Abstract

Roof-contact backfilling is a critical determinant of stope stability in cut-and-fill mining, and the rheological properties of backfill slurry decisively influence the quality of roof contact. To investigate the flow characteristics of unclassified tailings backfill slurry and their effect on rheological parameters, this study uses the Daye Iron Mine as its engineering case. It adopts a combined laboratory and numerical simulation approach. The physicochemical characteristics of the unclassified tailings and the rheological behavior of the slurry were systematically characterized using particle-size analysis, density measurements, spreadability tests, and rheometer measurements. Subsequently, a numerical model of the L-type flow tester was developed in COMSOL Multiphysics (6.4) to simulate the flow process at varying concentrations. Based on the simulation results, a Gaussian process regression (GPR)-based inversion model for rheological parameters was proposed, and the predictive performance of different kernel functions was compared and evaluated. Finally, the existing backfilling scheme at the Daye Iron Mine was optimized based on the obtained rheological characteristics to improve the roof-contact rate. The results indicate that the unclassified tailings from the Daye Iron Mine have a median particle size of 12.1 μm and a density of 2855 kg·m^−3^, with CaO, Al_2_O_3_, and MgO as the primary active components. Under the same cement-to-tailings ratio, slurry flowability decreases markedly with increasing concentration. The rheological curves exhibit three stages, with the third conforming to the Bingham model; both yield stress and viscosity increase exponentially with concentration. Evaluation of the inversion results demonstrates that the GPR model with the Rational Quadratic (RQ) kernel achieves optimal performance. The recommended slurry concentration for the Daye Iron Mine is determined to be in the range of 69–71%, and the recommended spacing between filling pipelines is 13.34–18 m. This study reveals the flow evolution patterns of unclassified tailings backfill slurry, demonstrates the potential of the GPR-based inversion approach, and optimizes the roof-contact backfilling scheme, offering a scientific reference for flow characterization and backfill optimization in analogous mining operations.

## 1. Introduction

Roof-contact control is a core process in cut-and-fill mining, as the quality of roof contact directly affects the stability of the mined-out area. Insufficient roof-contact ratios lead to excessive exposed roof areas, exceeding the self-stabilizing threshold of the rock mass and posing significant safety hazards [1,2,3,4]. The rheological properties of backfill slurry are among the key factors influencing roof-contact quality, with a negative coupling relationship existing between the flowability and strength of the slurry [5,6]. In mining practice, the strength requirements of the backfill body necessitate a sufficiently high solids concentration; however, the flowability of the slurry decreases with increasing concentration. Consequently, when the strength requirement is satisfied, the flowability of the slurry is often compromised, resulting in a low roof-contact ratio [7]. The flow characteristics of backfill slurry are governed by multiple factors and are primarily reflected in rheological parameters such as slump and yield stress [8,9,10].

Extensive research has been conducted on the rheological behavior of backfill slurry from three interconnected perspectives: laboratory experiments, rheological model development, and numerical simulation. In experimental studies, Xiao et al. [11] proposed an L-pipe flow method for rapid on-site yield stress estimation. Xiao et al. [12] found that slag-cemented paste backfill conforms to the Bingham model. Li et al. [13] confirmed solids concentration as the dominant factor governing rheological behavior. Yang et al. [14] investigated pipeline transport resistance and identified 68–72% as the favorable concentration range. Duan et al. [15] reported a negative correlation between concentration and flowability. Wang et al. [16] found no significant correlation between particle size and rheological properties. Hu et al. [17] observed nonlinear increases in yield stress and viscosity with concentration. Cao et al. [18] confirmed that yield stress and viscosity increase exponentially with concentration. Li et al. [19] found that high-concentration slurry exhibits distinct non-Newtonian behavior. Liu et al. [20] reported that ice-containing paste backfill conforms to the Bingham model. In terms of modeling methods, Li et al. [21] improved the slump prediction model with a correction factor. Ding et al. [22] found that the slurry conforms to the Bingham model at 70–74% but exhibits power-law behavior at other concentrations. Liang et al. [23] developed a regression model based on response surface methodology. Deng et al. [24] confirmed that the Bingham model adequately describes time-dependent rheological behavior. In numerical simulations, Tang et al. [25] derived that slurry accumulation follows a normal distribution. Shi et al. [26] observed a spatial gradient in particle size distribution along the flow path. Liu et al. [27] divided the stope flow process into four stages. Fang [28] investigated the flow and settlement of ice-containing slurry via CFD. Shi et al. [29] studied the effect of temperature on flowability and found that the slurry conforms to the Bingham model. However, most existing studies rely on either laboratory experiments or numerical simulations in isolation.

While the above studies have established a solid foundation for understanding the rheological behavior of backfill slurry, most of them rely on either laboratory experiments or numerical simulations in isolation. Consequently, three critical gaps remain unresolved: first, the flow evolution of backfill slurry under actual stope boundary conditions and flow path constraints has not been systematically elucidated; second, the quantitative relationships among slurry deposition morphology, self-flow extension distance, and roof-contact rate remain insufficiently characterized; third, a practical method for inverting rheological parameters from observable flow data is still lacking. To address these gaps, the present study takes the unclassified tailings backfill slurry of the Daye Iron Mine as the research object and adopts a combined approach of laboratory experiments and numerical simulation. The flow characteristics of the slurry are systematically investigated. The novelty of this study lies in the combination of the L-type flow tester and numerical simulation with Gaussian process regression for rheological parameter inversion. This approach is necessitated by the limitations of direct rheological measurements, which are time-consuming, costly, and cannot cover all possible operating conditions. Conventional regression methods, such as polynomial fitting and neural networks, either lack sufficient accuracy for small-sample high-dimensional problems or are prone to overfitting. In contrast, the GPR-based inversion framework leverages the rich flow-field data generated by numerical simulation and establishes a probabilistic mapping from observable flow variables to rheological parameters, offering advantages in uncertainty quantification and rapid estimation without repeated physical experiments. Finally, the existing backfilling scheme at the Daye Iron Mine is optimized to improve its roof-contact rate. The findings are expected to provide a scientific reference for mines with similar geological and backfilling conditions and to contribute to the refinement of roof-contact design methodologies in cut-and-fill mining operations.

## 2. Materials and Experimental Methods

### 2.1. Basic Physicochemical Properties of Backfill Slurry

#### 2.1.1. Particle Size Analysis of Unclassified Tailings

The particle size distribution of the unclassified tailings from the Daye Iron Mine was determined using a Mastersizer 3000 laser diffraction particle size analyzer (Malvern Panalytical, Malvern, UK), and the results are presented in Table 1.

The sorting coefficient *S*_0_ serves as a critical parameter for characterizing the particle size distribution of tailings. A value approaching unity indicates a uniform particle size distribution and well-sorted particles, whereas a higher value signifies a broader distribution and poor sorting. It should be noted that this index is conventionally used for continuously graded materials such as mine tailings; for discrete or bimodal distributions, this metric may not be physically intuitive and should be interpreted with caution. This coefficient is calculated using the following equation:

(1) $$S_{0}=\frac{d_{75}}{d_{25}}$$

where

*d*_75_—the particle size corresponding to 75% passing on the particle size distribution curve;

*d*_25_—the particle size corresponding to 25% passing on the particle size distribution curve.

**Figure 1:**
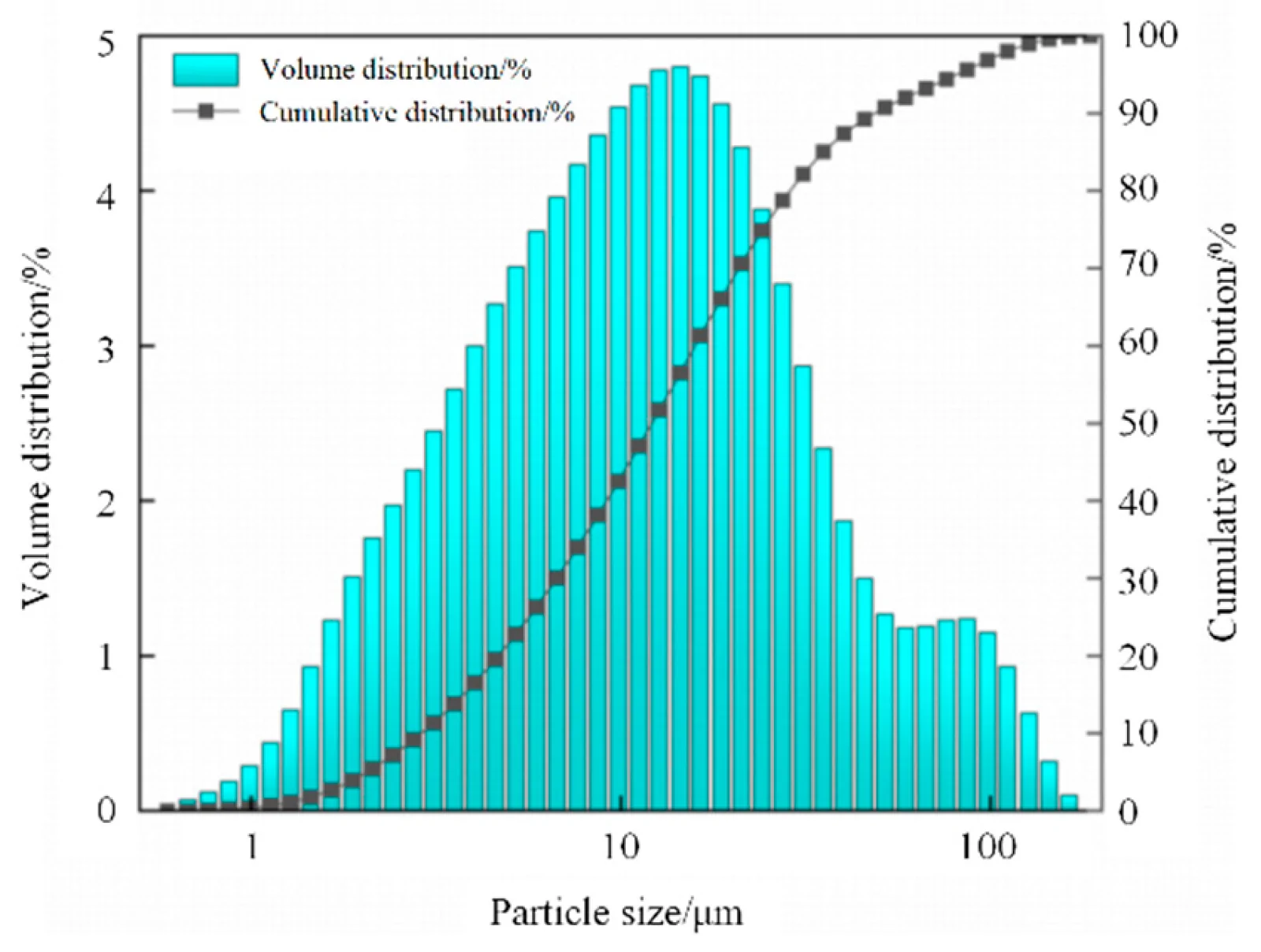
Particle size gradation analysis of unclassified tailings from the Daye Iron Mine.

Based on the measured particle size distribution data, the particle size distribution curve for the unclassified tailings from the Daye Iron Mine is presented in Figure 1.

From the particle size distribution curve, the characteristic particle sizes were obtained as *d*_25_ = 5.71 μm and *d*_75_ = 24.33 μm. Substituting these values into Equation (1) yields a sorting coefficient *S*_0_ = 4.26.

The median particle size *d*_50_ of the unclassified tailings from the Daye Iron Mine was found to be 12.1 μm, which corresponds to the 50% cumulative passing size on the distribution curve and characterizes the average particle size of the system. Given the fine median size and the high sorting coefficient (*S*_0_ > 4), the tailings are classified as ultra-fine and poorly graded.

#### 2.1.2. Density Analysis of Tailings

The density of the unclassified tailings from the Daye Iron Mine was determined using the pycnometer method [30]. First, the mass of the dry pycnometer was recorded as *K*_1_. Approximately 0.015 kg of the tailings sample was then placed into the pycnometer, and the combined mass of the pycnometer and tailings was recorded as *K*_2_. Distilled water was added to the pycnometer until it reached approximately half of its volume, and the pycnometer was heated to expel dissolved gases from the water. After cooling to a constant temperature of 20 °C, distilled water was precisely added dropwise to the calibration mark using a dropper, and the total mass of the pycnometer, tailings, and water was recorded as *K*_3_. Finally, the contents of the pycnometer were discarded, and the pycnometer was thoroughly cleaned. Distilled water was refilled to the same calibration mark, and the mass of the pycnometer and water was recorded as *K*_4_.

The density of the tailings was calculated using the following formula:

(2) $$G_{s}=\frac{K_{2}-K_{1}}{K_{2}-K_{1}-K_{3}+K_{4}}G_{m}$$

where

*G_m_*—density of distilled water at 20 °C (998 kg·m^−3^);

*G_s_*—density of the tailings, kg·m^−3^.

Three parallel tests were conducted to ensure the reliability and accuracy of the measurements, and the average value was taken as the density of the unclassified tailings. The experimental results are presented in Table 2.

Based on the experimental analysis, the density of the unclassified tailings from the Daye Iron Mine was determined to be 2855 kg·m^−3^.

#### 2.1.3. Chemical Composition Analysis of Tailings

**Figure 2:**
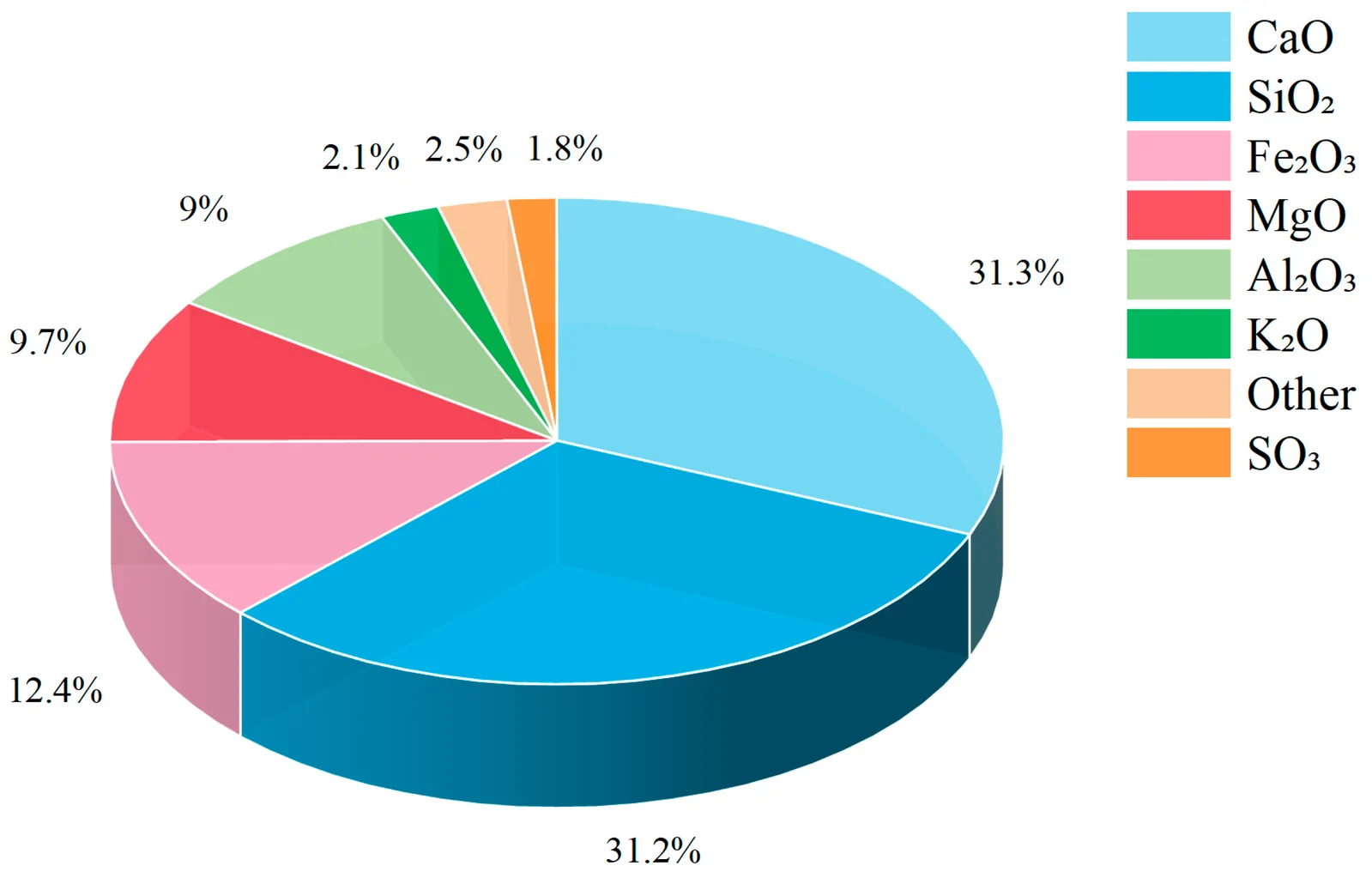
Major chemical components of unclassified tailings from the Daye Iron Mine.

A representative tailings sample was taken from the slurry at the processing plant, thoroughly mixed, and dried to obtain a composite sample. The major chemical components of the unclassified tailings from the Daye Iron Mine were then determined by X-ray fluorescence (XRF) analysis, and the results are presented in Figure 2.

As shown in Figure 2, CaO, Al_2_O_3_, SiO_2_, and MgO are the predominant oxide components commonly found in unclassified tailings, accounting for 81.19% of the total mass. Among these, CaO (31.28%), Al_2_O_3_ (8.99%), and MgO (9.70%) are reactive oxides that promote oxidation reactions and the development of cementitious networks, thereby enhancing the strength of the backfill body. In contrast, SiO_2_ (31.21%) is an inert oxide that inhibits the growth of cementitious networks; however, it exhibits favorable sedimentation performance and permeability. The sulfur content is only 1.84%, indicating a low sulfide content that has a negligible effect on the strength of the backfill body.

#### 2.1.4. Density of Backfill Slurry

The density of the backfill slurry is expressed as follows:

(3) $$P_{m}=\frac{P_{c}G_{s}(1+m)}{H_{w}\left(G_{s}+mP_{c}\right)+\left(1-H_{w}\right)[P_{c}G_{s}\left(1+m\right)]}$$

where

*P_m_*—the density of the backfill slurry, t/m^3^;

*m*—the cement-to-tailings ratio;

*H_w_*—the solids concentration, %;

*P_c_*—the density of the cementitious material, t/m^3^.

The density of the dry solid mixture is given by:

(4) $$P_{s}=\frac{P_{c}G_{s}(1+m)}{P_{c}+mG_{s}}$$

where

*P_s_*—the density of the dry solid mixture, t/m^3^.

The densities of the backfill slurry at different solids concentrations were calculated using Equations (3) and (4), and the results are presented in detail in Table 3.

### 2.2. Slump Flow of Unclassified Tailings Backfill Slurry

**Figure 3:**
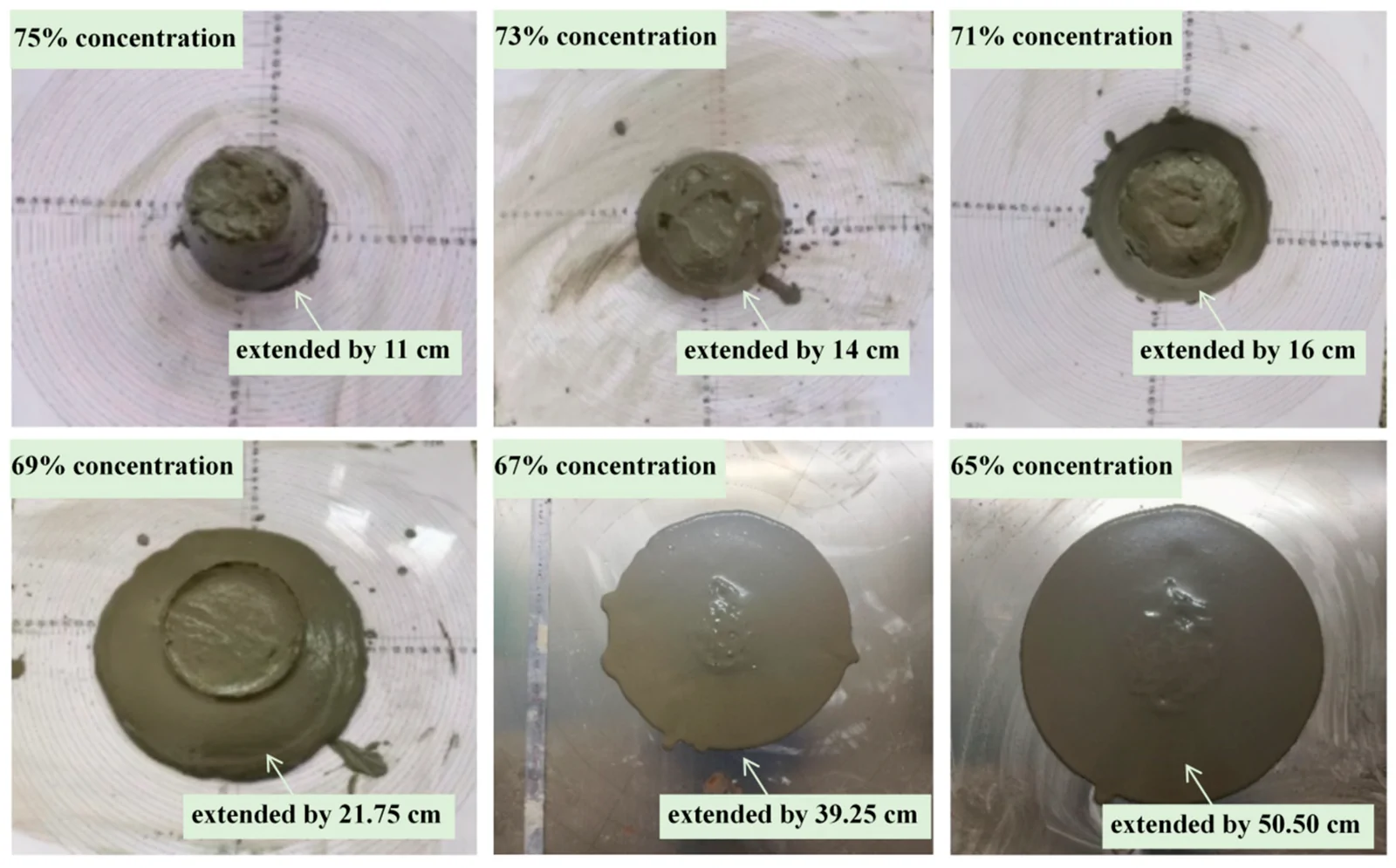
Spreadability test results.

Slump flow tests of the unclassified tailings backfill slurry were conducted using an Abrams cone, with cementitious powder as the binder. Backfill slurries with varying solids concentrations and cement-to-tailings ratios were prepared for testing. The binder used in this study is the cementitious material employed on-site at the Daye Iron Mine (mainly ordinary Portland cement). For slurry preparation, the cementitious material, tailings, and water were first weighed according to the mix proportion; the dry materials were then placed into a mixing bowl and mixed evenly before the addition of water, followed by continuous mixing for 3–5 min. All tests were conducted at room temperature. During the tests, the slurries with solids concentrations of 65% and 67% flowed beyond the edge of the slump flow plate, rendering the measurements invalid. Consequently, a larger slump flow plate was adopted for the subsequent two test groups, as illustrated in Figure 3.

Although different slump flow plates were used in the tests, both plates had smooth surfaces. Therefore, the influence of surface roughness on the test results was considered negligible. In each test, both the lateral and longitudinal spread distances were measured, and their average was taken as the spreadability value to eliminate possible anisotropy effects. It should be noted that the spreadability tests were performed once for each mix proportion; the lateral and longitudinal measurements were averaged to account for flow asymmetry, rather than as experimental replicates. The slump flow test results of the unclassified tailings backfill slurry are presented in Table 4.

As shown in Table 4, at a constant cement-to-tailings ratio, the flowability of the backfill slurry decreased as the solids concentration increased. When the solids concentration exceeded 69%, the flowability deteriorated significantly, and the slurry transitioned from a liquid to a paste-like state. At 75% concentration, the slurry exhibited virtually no flow, with an average slump flow of only 11.00 cm. In contrast, the slurries with solids concentrations of 65% and 67% exhibited excellent flowability, with average slump flow values of 50.50 cm and 39.25 cm, respectively, significantly greater than that of the 75% concentration slurry.

### 2.3. Experimental Study on Flow Characteristics of Unclassified Tailings Backfill Slurry

Rheological parameters are core indicators characterizing the flow behavior of backfill slurry, directly influencing pipeline transportation and stope backfilling performance. The primary rheological parameters of backfill slurry are yield stress and plastic viscosity. Yield stress represents the minimum shear stress required to initiate flow from a static state, while viscosity reflects the internal frictional resistance during slurry flow [31,32,33,34]. In fluid mechanics, rheological models are broadly classified into Newtonian and non-Newtonian fluids. Cemented unclassified tailings backfill slurry is a non-Newtonian fluid, and the most commonly used rheological models for its characterization are the Bingham model and the Herschel–Bulkley (H–B) model [35,36,37]. The mathematical expressions of these rheological models are presented in Table 5.

Based on the slump flow test results, the slurries with solids concentrations of 73% and 75% at a cement-to-tailings ratio of 1:4 exhibited extremely low flowability. Consequently, rheological measurements for these two concentrations were considered of limited value. Accordingly, backfill slurries with a cement-to-tailings ratio of 1:4 and solids concentrations of 65%, 67%, 69%, and 71% were prepared for rheological testing. Rheological measurements were performed using an Anton Paar MCR 302 rheometer (Anton Paar GmbH, Graz, Austria) with a concentric cylinder system and a gap of 1 mm between the rotor and the outer cylinder. No pre-shear was applied before testing. A full factorial experimental design was adopted, with the shear rate set in the range of 0.1–220 s^−1^, and the testing temperature was maintained at 20 ± 2 °C. The instrument was calibrated following the manufacturer’s standard procedures before use. It should be noted that the rheological measurements were performed once for each mix proportion; during a single rheometer run, the instrument automatically collected data points across the programmed shear rate range, with each point representing a stable averaged value from multiple samplings at the corresponding shear rate. These multiple readings within a single run are not intended as a substitute for independent experimental replication or as a measure of experimental variability.

**Figure 4:**
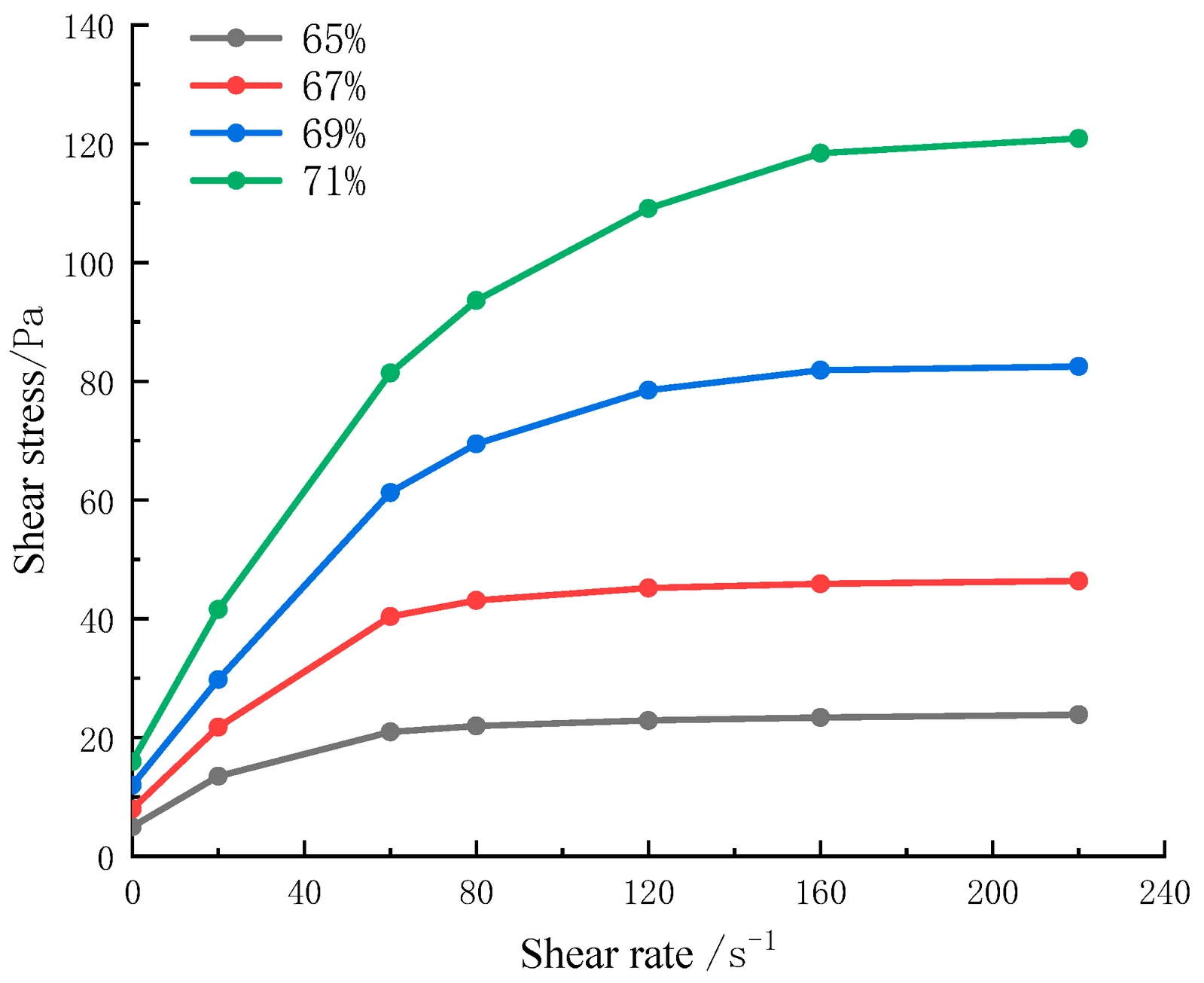
Apparent shear stress vs. shear rate curves of unclassified tailings backfill slurry at different concentrations.

After processing the experimental data using Origin (Pro 2021) software, the shear stress–shear rate curves for the backfill slurry at different solids concentrations are shown in Figure 4. (Selected representative data points are shown for clarity; the rheometer collected substantially more data points than plotted.)

As shown in Figure 4, the shear stress exhibited a positive nonlinear relationship with the shear rate, and the evolution of the rheological curves could be divided into three characteristic stages. In the initial stage, the shear stress rose sharply from zero to a peak value upon the onset of shear, after which it increased linearly with the shear rate. The frictional resistance generated by the interactions between the cementitious binder and the tailings particles hindered the rotation of the rheometer cylinder. To transition from a static to a flowing state, the slurry had to overcome this frictional resistance, with higher resistance leading to greater shear stress at the onset of flow. Consequently, slurries with higher solids concentrations exhibited higher viscosity and shear stress at the initiation of flow. In the second stage, the shear stress continued to increase nonlinearly with the shear rate, but the rate of increase gradually diminished. Higher solids concentrations were associated with a more pronounced increase in the rate of shear stress growth. In the third stage, the rate of increase in shear stress became constant, and the relationship between shear stress and shear rate approached a nearly linear trend, indicating that the rheological curve exhibited typical Bingham fluid characteristics of a non-Newtonian fluid.

In summary, the unclassified tailings backfill slurry exhibited pseudoplastic rheological behavior with a yield stress. At high shear rates, the rheological curves provided a more representative depiction of the flow characteristics of the slurry. Since the slurry in pipeline transportation and stope flow predominantly experiences medium-to-high shear rates, this study focuses on the third-stage (high-shear-rate) regime for parameter quantification. Therefore, based on the experimental rheological data, the data from the third stage of the rheological curves were selected, and the yield stress and plastic viscosity parameters of the Bingham model were determined through data fitting. The fitted results are presented in Table 6.

**Figure 5:**
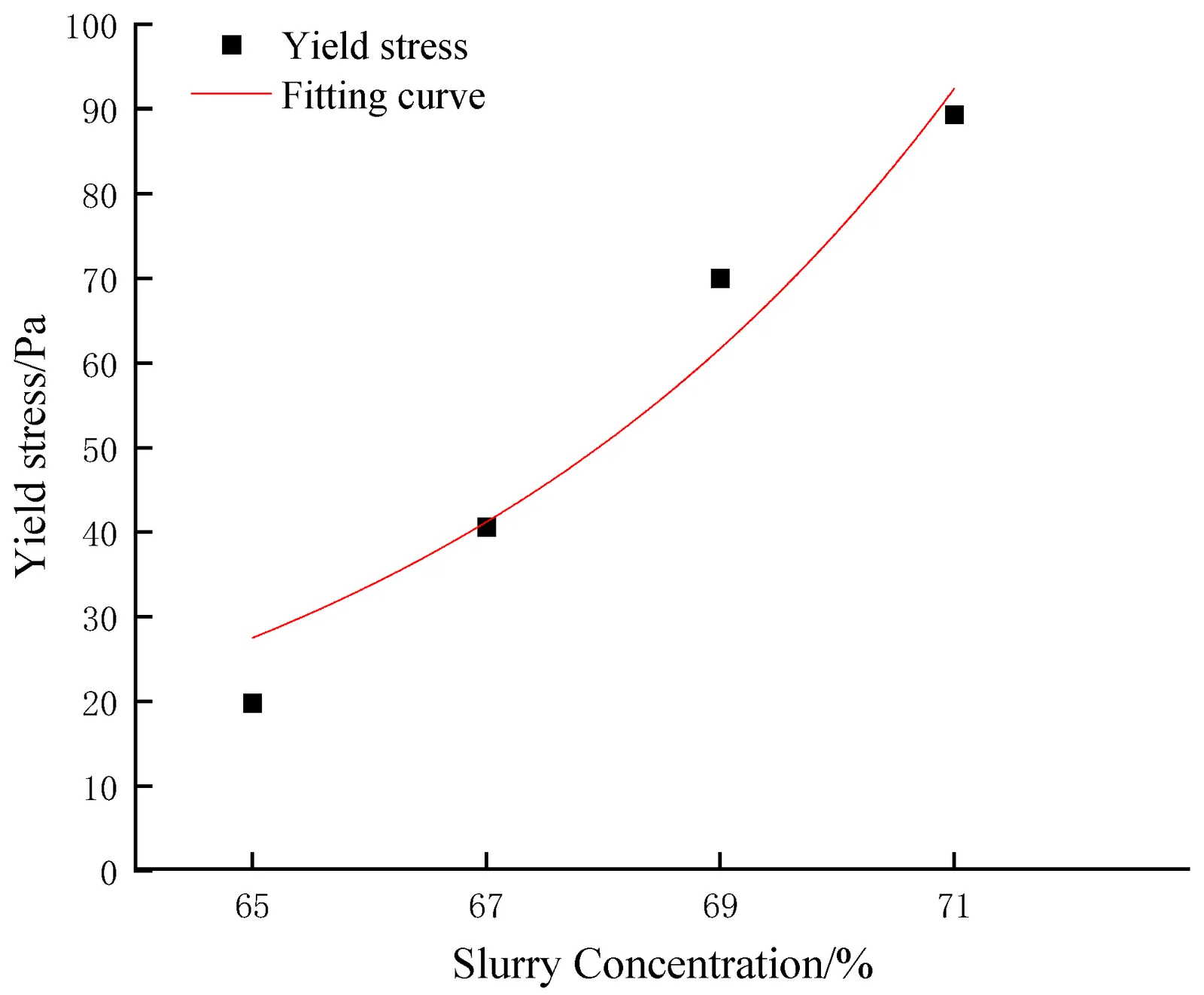
Fitting curves of dynamic yield stress vs. slurry solids concentration.

Based on the fitting results presented in Table 6, a regression analysis was performed between the solids concentration and yield stress of the four groups of unclassified tailings backfill slurry. The fitting curve of the regression equation is shown in Figure 5, and the regression equation is expressed as follows:

(5) $$\tau_{d}=5.498\times 10^{-5}\times 1.224^{C_{w}}\left(R^{2}=0.9273\right)$$

where

*τ_d_*—the dynamic yield stress: the yield stress derived from dynamic rheological measurements, Pa;

*c_w_*—the solids concentration by mass, %.

As shown in Figure 5, the yield stress increased approximately exponentially with increasing solids concentration.

**Figure 6:**
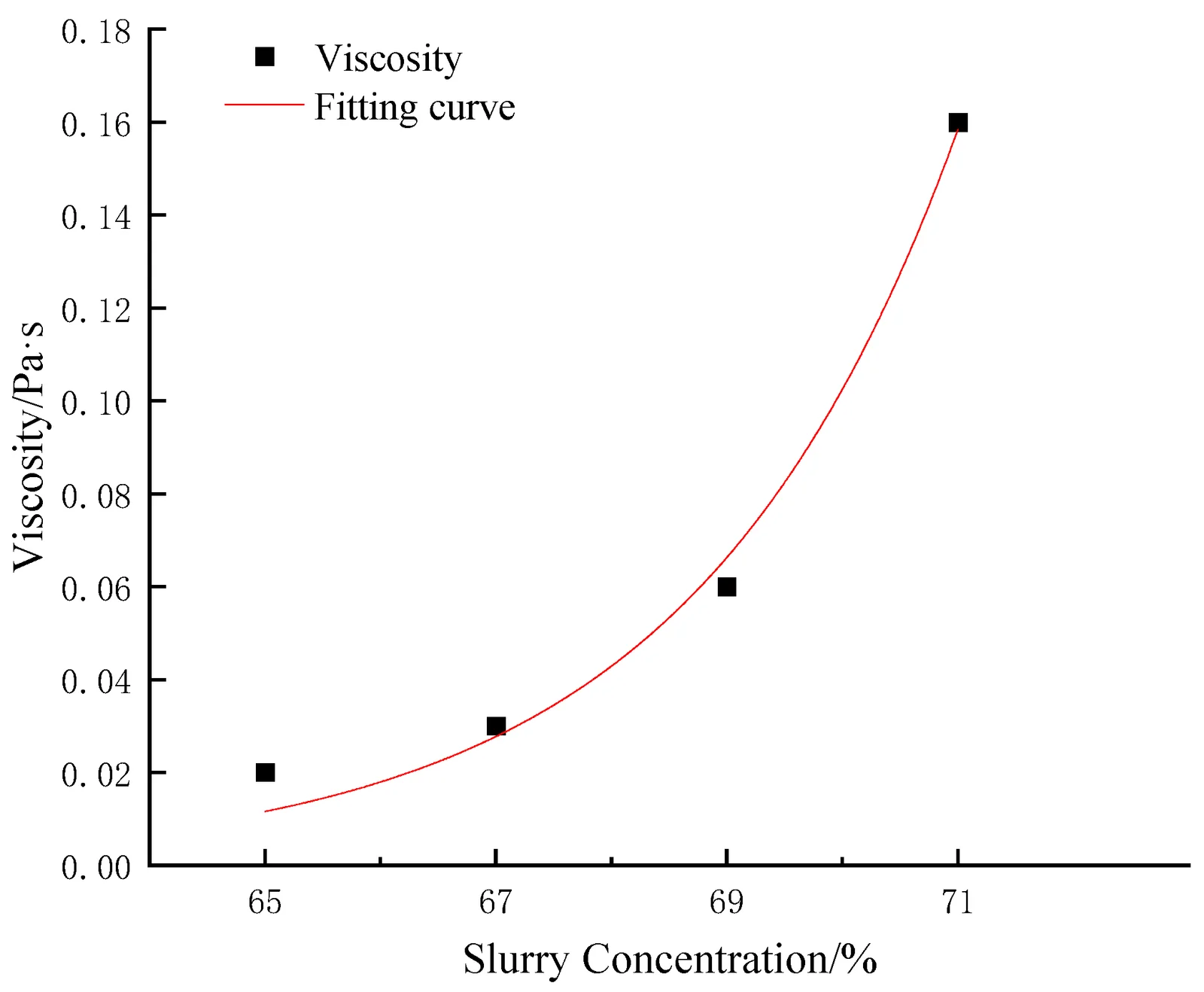
Fitting curves of dynamic viscosity vs. slurry solids concentration.

Based on the fitting results presented in Table 6, a regression analysis was performed by fitting the solids concentration against the plastic viscosity for the four groups of unclassified tailings backfill slurry. The fitted curve is shown in Figure 6, and the regression equation is expressed as follows:

(6) $$\mu^{\prime }=5.925\times 10^{-15}\times 1.869^{c_{w}}\left(R^{2}=0.9856\right)$$

where

$\mu^{\prime }$—the dynamic viscosity: the viscosity derived from dynamic rheological measurements, Pa·s.

As illustrated in Figure 6, the viscosity also increased approximately exponentially with increasing solids concentration. From the data in Table 6, it can be observed that the viscosity values were very low at solids concentrations of 65–67%. At 67% concentration, the viscosity began to increase gradually, and at 71% concentration, it increased substantially, indicating a significant enhancement in the viscous properties of the slurry at higher solids concentrations.

## 3. Numerical Simulation of Flow Characteristics of Unclassified Tailings Backfill Slurry

### 3.1. L-Type Flow Tester Setup

When the backfill slurry is discharged into the stope, it spreads outward under gravity. Upon reaching the stope boundaries, the slurry accumulates upward and forms a specific deposition pattern due to its internal resistance, creating a self-flow slope relative to the horizontal plane. The flowability of the backfill slurry is a key factor governing the self-flow slope [38]. In laboratory tests, the flow behavior of the slurry in the horizontal channel of the L-type flow tester closely resembles that in an actual stope. Therefore, the L-type flow tester serves as an effective tool for analyzing the flow characteristics of backfill slurry in stopes.

**Figure 7:**
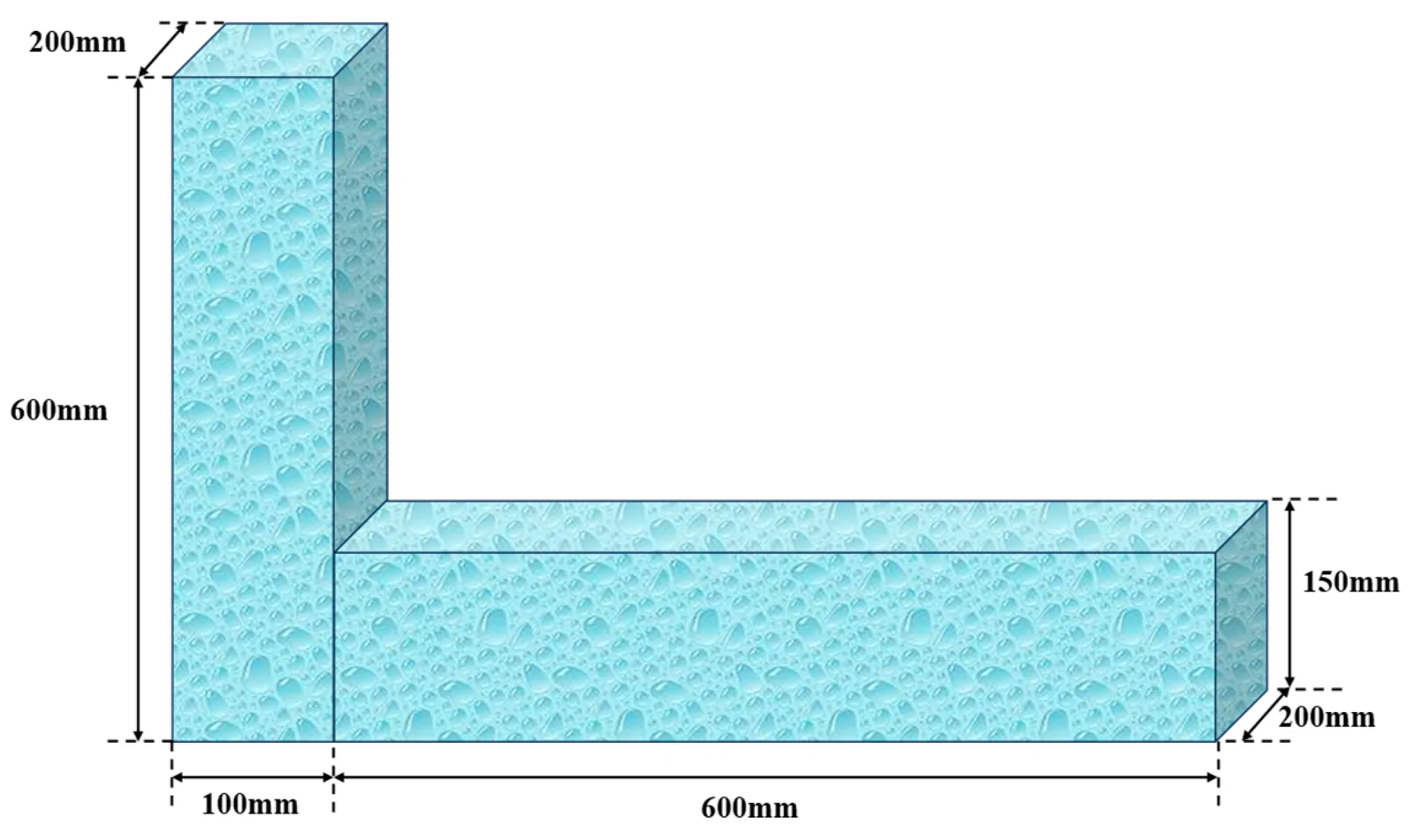
L-Type Flow Tester Schematic Diagram.

The L-type flow tester consists of a vertical section with a length of 100 mm and a height of 600 mm, and a horizontal section with a length of 600 mm, a width of 200 mm, and a height of 150 mm, as illustrated in Figure 7. Given the large volume of slurry required for physical testing, the flow behavior of the backfill slurry in the L-type flow tester was numerically simulated using COMSOL Multiphysics (6.4) software.

### 3.2. Numerical Simulation Parameters Settings

In this numerical simulation, the backfill slurry was treated as a homogeneous fluid due to the unclassified nature of the tailings, and the effect of temperature on the flow behavior was neglected. To capture the free surface evolution of the slurry during flow, which involves a separated multiphase flow problem, the two-phase flow with the level set method was selected as the physical interface in COMSOL Multiphysics (6.4).

**Figure 8:**
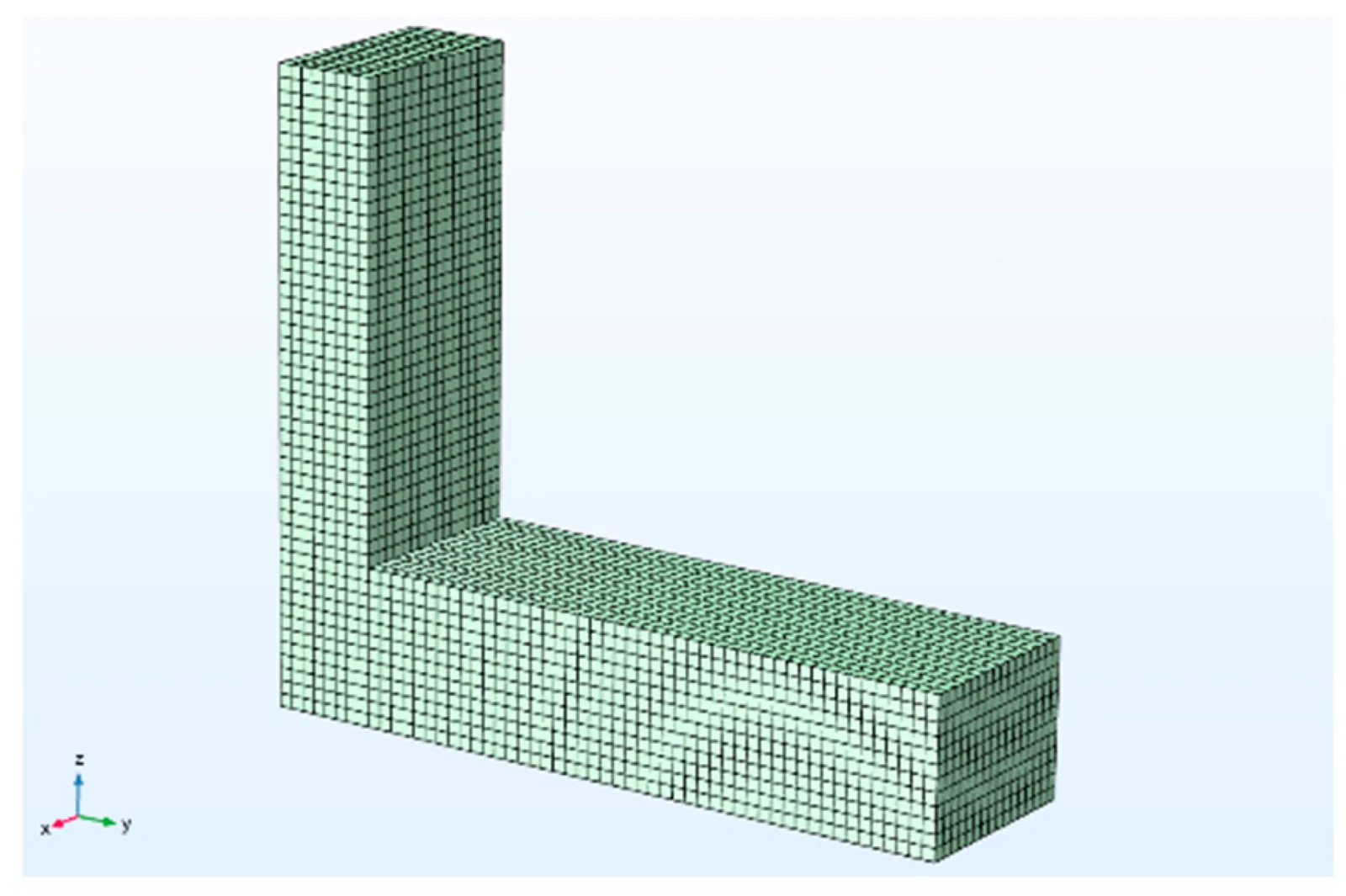
The numerical simulation mesh diagram.

To ensure the accuracy of the simulation results, the model was discretized using a structured hexahedral mesh with 15,168 elements, achieving a minimum element quality of 0.995 and an average element quality of 0.9999, as shown in Figure 8. Mesh-independence was verified by refining the mesh by approximately 30%, which yielded a deviation of less than 2% in the simulation results; time-step sensitivity was assessed by reducing the time step from 0.01 s to 0.005 s, with a deviation of less than 3%. The boundary conditions were set as the pressure inlet and pressure outlet, with the walls defined as wetted walls. The inlet pressure is hydrostatic pressure, and the outlet is set to atmospheric pressure (0 Pa gauge). A contact angle of π/2 rad is specified for the wetted walls, and surface tension is neglected as gravitational forces dominate the slurry flow. The two phases were specified as the backfill slurry and air, with the slurry modeled as a Bingham fluid. The Bingham constitutive behavior is implemented using the Papanastasiou regularization method with a regularization parameter of mp = 10^−6^ s. The solver was set to transient mode using the PARDISO algorithm with a relative tolerance of 0.01 as the convergence criterion, and a time step of 0.01 s was adopted. The specific parameters of the backfill slurry used in the simulation are listed in Table 7.

### 3.3. Analysis of Numerical Simulation Results

**Figure 9:**
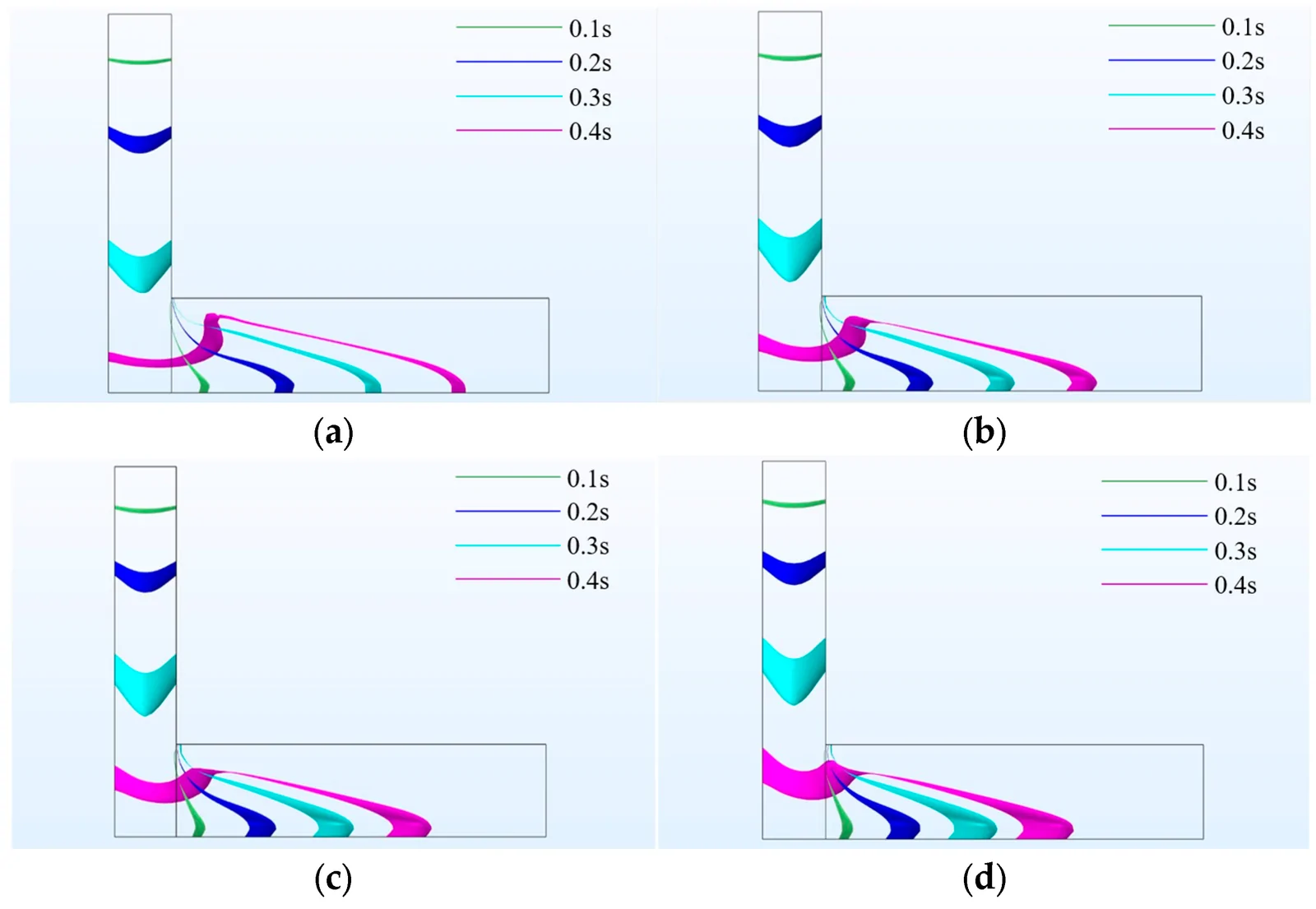
Plot of Total Synchronous Time Isosurface of Multiple Mass Concentrations of Tailings Backfill Slurry: (**a**) 65% Slurry Concentration; (**b**) 67% Slurry Concentration; (**c**) 69% Slurry Concentration; (**d**) 71% Slurry Concentration.

Numerical simulations of the L-type flow tester were conducted using COMSOL Multiphysics (6.4). The mid-section of the L-type flow tester was selected to generate contour maps illustrating the flow trajectory of the backfill slurry at four different solids concentrations at the same time instant, as shown in Figure 9.

As can be observed from Figure 9, the slurry with a solids concentration of 65% had already flowed to approximately 450 mm along the horizontal channel at 0.4 s, whereas the slurry with a concentration of 71% had not yet reached 400 mm by the same time, indicating that solids concentration significantly influenced the flow velocity of the slurry. With increasing concentration, both yield stress and plastic viscosity increased, resulting in a slower flow velocity in the horizontal channel and an expansion of the shear zone.

**Figure 10:**
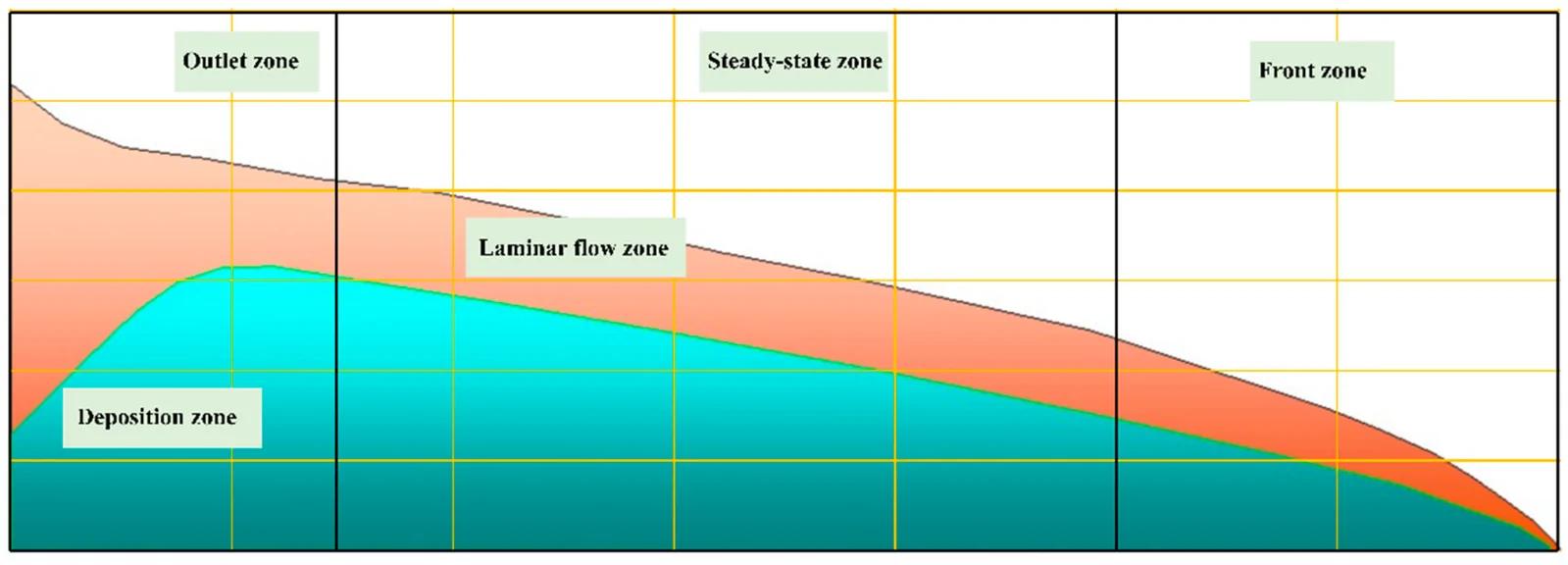
Flow diagram of filling slurry.

By analyzing the flow regimes in the four test groups, the flow pattern of the backfill slurry in the horizontal channel could be divided into three zones, the outlet zone, the steady-state zone, and the flow front zone, as illustrated in Figure 10. At the onset of flow, gravitational forces drove the slurry to move along the horizontal channel. In the outlet zone, a local rise in the slurry free surface is observed, which may be attributed to the conversion between kinetic and potential energy caused by the abrupt change in flow direction. In the steady-state zone, as the flow distance increased, gravitational potential energy decreased, and the slurry flowed slowly under gravity, forming a laminar flow regime. At the flow front, shear interaction occurred between the slurry and the container boundaries, continuously supplying fluid for shear layer development.

**Figure 11:**
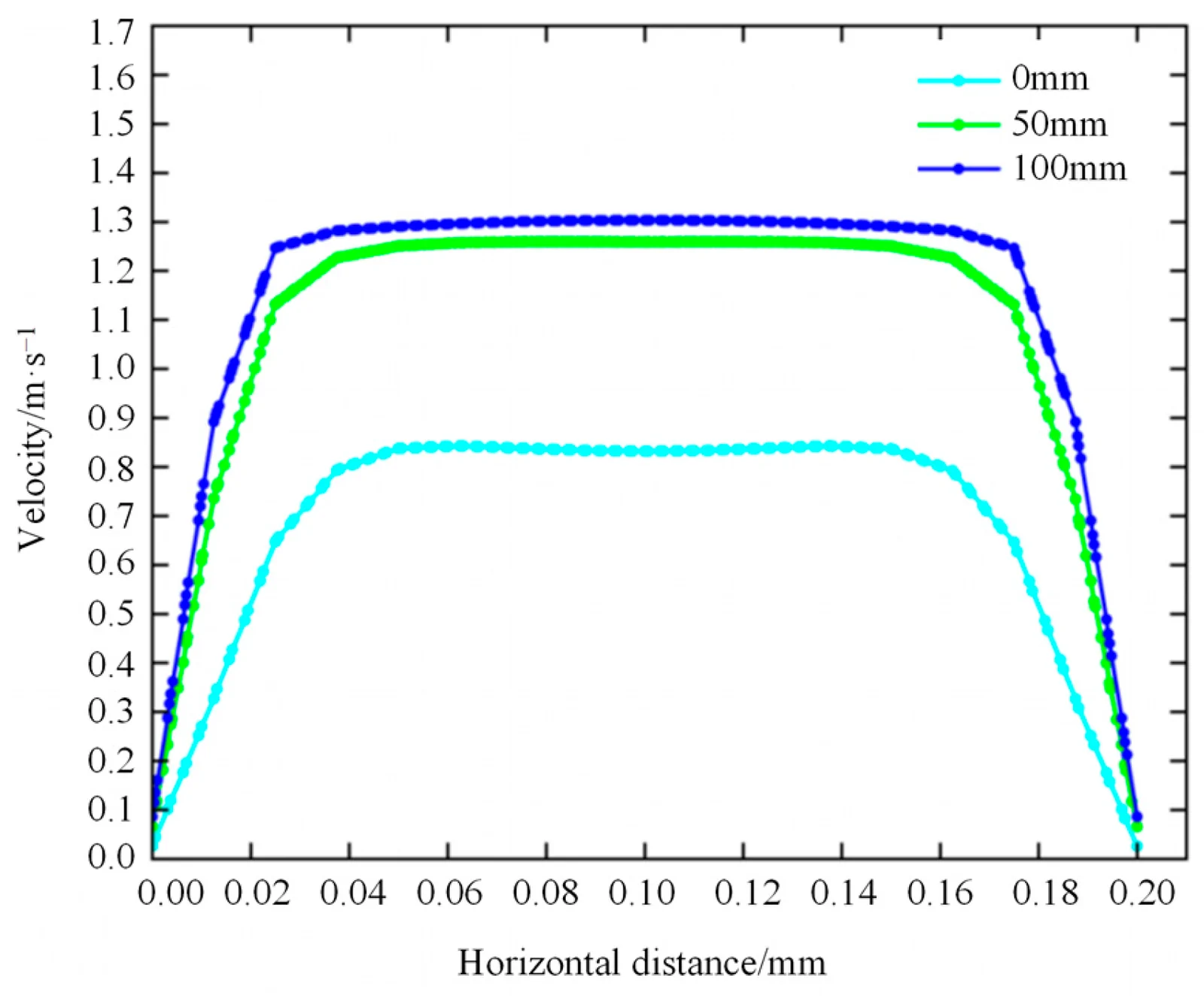
Velocity Distribution Plot.

To investigate the velocity distribution characteristics of the slurry in the horizontal direction, the local velocity field within the flow domain is denoted as *u*, while the monitored velocities at specific positions along the horizontal channel are denoted as *V*_1_ to *V*_5_. The velocity profiles at the outlet and at positions 50 mm and 100 mm downstream from the outlet were extracted from the post-processing results of the numerical simulation at 0.2 s for the 71% concentration slurry, as shown in Figure 11. It can be observed from Figure 11 that the local velocity *u* remained constant at the center of the horizontal channel, forming a plug flow region. Moving from the center toward the wall, the velocity *u* remained constant until a certain position, after which it began to decrease; this region of decreasing velocity gradient is defined as the shear zone. The local velocity *u* increased with increasing distance from the outlet, but the gradient of velocity increase diminished with distance.

## 4. Rheological Parameter Inversion Based on Gaussian Process Regression

### 4.1. Extraction of Flow-State Data

In the numerical simulations of the L-type flow tester, the flow time, flow velocity, and free surface elevation of the backfill slurry in the horizontal channel exhibited correlations with its yield stress and viscosity. Therefore, from a theoretical standpoint, the rheological parameters of the backfill slurry can be inversely determined using the post-processing data obtained from the numerical simulations.

Based on the rheological parameters and density design schemes from the aforementioned numerical simulations, a new experimental design was developed for the inversion analysis. An orthogonal experimental design was adopted with three factors (yield stress, viscosity, and density) at five levels each. The L25 (56) orthogonal array was selected, resulting in 25 simulation cases, as presented in Table 8.

The horizontal channel of the L-type flow tester was divided into five segments, each 100 mm in length. The times required for the slurry to reach positions of 100 mm, 200 mm, 300 mm, 400 mm, and 500 mm along the horizontal channel were recorded as *T*_1_, *T*_2_, *T*_3_, *T*_4_, and *T*_5_, respectively. The corresponding flow velocities at these positions were denoted as *V*_1_, *V*_2_, *V*_3_, *V*_4_, and *V*_5_. The inclination angles of the slurry free surface over the intervals of 0–100 mm, 100–200 mm, and 200–300 mm at 0.35 s were recorded as *δ*_1_, *δ*_2_, and *δ*_3_, respectively. Numerical simulations of the orthogonal experimental design were performed using COMSOL Multiphysics (6.4), and the post-processing results are presented in Table 9.

### 4.2. Gaussian Process Regression and Hyperparameter Optimization

Given that the dataset in this study is characterized by a small sample size, high-dimensional feature space, and complex nonlinear relationships among variables, the Gaussian process regression (GPR) method was employed for the inversion analysis of rheological parameters. As a non-parametric approach, GPR is capable of capturing nonlinear mappings in high-dimensional spaces while mitigating the risk of overfitting associated with small-sample datasets. Its core principle is to define a probabilistic model for predicting function values and quantifying the associated prediction uncertainty [39,40,41].

In the inversion prediction framework, the training dataset is denoted as D = (A, B), where the input feature vector *a* = (*a*_1_, *a*_2_, *a*_3_, …, *a_i_*) (with *i* = n) comprises the rheological parameters of the backfill slurry (*T*, *V*, *δ, P*), and the corresponding output feature vector *b* = (*b*_1_, *b*_2_, *b*_3_, …, *b_i_*) (with *i* = n) comprises the yield stress *τ* and plastic viscosity *η*. The dataset is assumed to be generated by an unknown function *f*(*a*), and the prior distribution of the target variables follows a Gaussian distribution, with *f*(*a*) drawn from a Gaussian process *GP*(*m*(*a*), *k*(*a*, *a*′)). The mean function *m*(*a*) represents the expected value of *f*(*a*) and is typically set to zero as the prior. The kernel function *k*(*a*, *a*′), which measures the correlation between any two points and *a*′, is a key component that determines the shape of the prior and posterior distributions of the Gaussian process. Commonly used kernel functions include the RBF kernel, the Matérn kernel, and the rational quadratic (RQ) kernel [42,43].

(7) $$m\left(a\right)=E\left(f\left(a\right)\right)$$

(8) $$k\left(a,a^{\prime }\right)=E\left[\left(f\left(a\right)-m\left(a\right)\right)\left(f\left(a^{\prime }\right)-m\left(a^{\prime }\right)\right)\right]$$

where

*a* and *a*′—arbitrary random variables.

According to the properties of the multivariate Gaussian distribution, the prediction results for the dataset follow a Gaussian distribution. For a predicted value *b*′, the estimated mean $\mu_{b}$ and variance $\eta_{b}$ are given by:

(9) $$\mu_{b}=k(a_{\#},a){\left[k(a,a)+\sigma_{n}^{2}I_{n}\right]}^{-1}b$$

(10) $$\eta_{b}=k(a_{\#},a_{\#})-k(a_{\#},a){\left[k(a,a)+\sigma_{n}^{2}I_{n}\right]}^{-1}k(a,a_{\#})$$

where

*a*—the input variable of the training dataset;

*a*_#_—the input variable of the test sample;

*b*—the target variable of the training set;

*k*(*a*_#_, *a*_#_)—the covariance matrix of *a*_#_;

*k*(*a*_#_, *a*) and *k*(*a*, *a*_#_)—the *n* × 1 covariance matrices between *a* and *a*_#;_

*k*(*a*, *a*)—the *n* × *n* symmetric positive definite covariance matrix;

$\sigma_{n}^{2}$—the Gaussian white noise variance;

$I_{n}$—the *n*-dimensional identity matrix.

The GPR model is non-parametric, and its predictive performance is influenced by the hyperparameters of the kernel function, such as length scale and signal variance. Different hyperparameter values lead to different prediction outcomes. Optimizing these hyperparameters ensures that the model effectively captures the underlying relationships in the data, thereby improving prediction accuracy. Since the posterior distribution of the hyperparameters in GPR is difficult to compute directly, the log marginal likelihood (LML) is commonly used to evaluate the agreement between the model and the data. Maximizing the LML balances model complexity and data fitting, thereby preventing overfitting or underfitting. The LML is maximized using the quasi-Newton method to explore the optimal hyperparameters that maximize the likelihood of the test samples. The LML function is expressed as follows:

(11) $$\mathrm{lg}P(y|x,\theta )=-\frac{1}{2}y^{T}{\left(K+\sigma_{n}^{2}I\right)}^{-1}y-\frac{1}{2}\mathrm{lg}\left|K+\sigma_{n}^{2}I\right|-\frac{n}{2}\mathrm{lg}(2\pi )$$

where

*θ*—the hyperparameters of the kernel function;

*n*—the number of samples.

**Figure 12:**
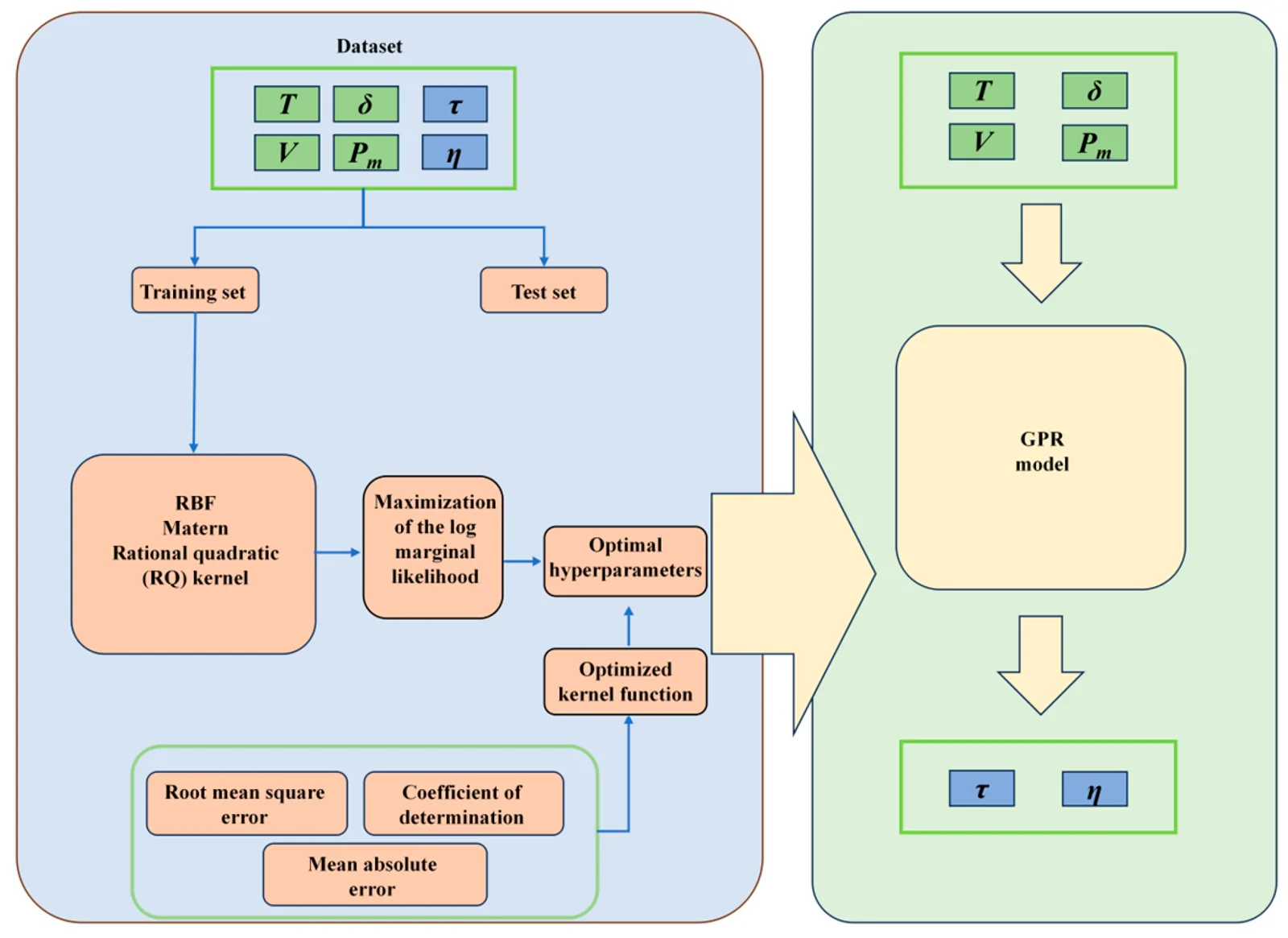
Prediction Flowchart.

The specific workflow of the hyperparameter-optimized GPR-based inversion method for rheological parameters of backfill slurry is illustrated in Figure 12.

As shown in Figure 12, the inversion prediction procedure begins with the collection of rheological parameter datasets. The input variables include *T*_1_, *T*_2_, *T*_3_, *T*_4_, *T*_5_, *V*_1_, *V*_2_, *V*_3_, *V*_4_, *V*_5_, *δ*_1_, *δ*_2_, *δ*_3_, and the density of the backfill slurry, while the output variables are *τ* and *η*. The dataset is then normalized and divided into training and test sets. Kernel function selection and hyperparameter optimization are performed using 5-fold cross-validation exclusively on the training set. The test set is strictly reserved solely for final model performance evaluation and is not involved in any stage of model training, kernel selection, or hyperparameter optimization. The training set is used to build the GPR model, and the optimal hyperparameters are explored by maximizing the LML. The optimized hyperparameters are then applied to the model for prediction and evaluation on the test set, yielding the predicted mean and variance of the rheological parameters for the unclassified tailings backfill slurry. The predicted results from the GPR model are compared with the measured values, and the optimal kernel function is selected based on performance metrics such as mean squared error (MSE), mean absolute error (MAE), and coefficient of determination (*R*^2^). Finally, for new samples with input variables *T*_1_, *T*_2_, *T*_3_, *T*_4_, *T*_5_, *V*_1_, *V*_2_, *V*_3_, *V*_4_, *V*_5_, *δ*_1_, *δ*_2_, *δ*_3_, and slurry density, the pre-trained GPR model is used to estimate the rheological parameters of the unclassified tailings backfill slurry.

### 4.3. Preprocessing of Experimental Data

To address dimensional differences and varying feature ranges in the dataset, a normalization method was applied during data preprocessing. Normalization ensures that all features have the same value range, preventing any single feature from dominating the model and enabling more effective hyperparameter optimization.

Data normalization is a critical preprocessing step in machine learning. In this study, the min–max normalization method was adopted to preprocess the dataset. The normalized independent and dependent variables of the flow-state parameters of the backfill slurry are presented in Table 10 and Table 11, respectively.

### 4.4. Analysis of Inversion Results

The performance of the regression models was evaluated using several metrics, including mean squared error (MSE), root mean squared error (RMSE), mean absolute error (MAE), and coefficient of determination (*R*^2^) [44]. In this study, the prediction performance of the backfill slurry models was assessed using MAE, MSE, and *R*^2^. The MSE is calculated as follows:

(12) $$\mathrm{MSE}=\frac{1}{n}\sum_{i=1}^{n}{\left(u_{i}-{\hat{u}}_{i}\right)}^{2}$$

where

$u_{i}$—actual value;

${\hat{u}}_{i}$—predicted value.

The coefficient of determination (*R*^2^) is calculated as:

(13) $$R^{2}=1-\frac{\sum_{i=1}^{n}{\left(u_{i}-{\hat{u}}_{i}\right)}^{2}}{\sum_{i=1}^{n}{\left(u_{i}-\bar{u}\right)}^{2}}$$

where

$\bar{u}$—mean of the actual values.

The mean absolute error (MAE) is calculated as:

(14) $$\mathrm{MAE}=\frac{1}{n}\sum_{i=1}^{n}\left|{\hat{u}}_{i}-u_{i}\right|$$

To mitigate the risk of overfitting associated with the limited sample size (n = 25 cases, 14 input variables), repeated 5-fold cross-validation (5 splits × 5 repeats) and leave-one-out cross-validation (LOOCV, 25 folds) were performed on the training set. The validation results are summarized in Table 12 and Table 13. The cross-validation results indicate that the RQ kernel achieves the most balanced performance for both targets. Under LOOCV, the RQ kernel attained the lowest mean squared error for both yield stress and viscosity coefficient, tied with RBF, while the Matern kernels consistently underperformed for viscosity inversion. Therefore, based on the cross-validation results, the RQ kernel is selected as the preferred kernel for the GPR-based inversion model.

A limitation of the cross-validation results is that the predictive performance for viscosity coefficient inversion is substantially lower than that for yield stress. This discrepancy is attributed to the narrow range of viscosity values in the dataset and the relatively low sensitivity of the L-type flow tester features to viscosity variations under the present numerical conditions. Therefore, the viscosity predictions should be interpreted with caution, and further experimental validation is recommended before applying them in engineering design.

The performance of the three kernel functions on the test set was evaluated using MATLAB (R2024a), and the results are summarized in Table 14. These test-set results serve only as a final performance assessment and were not used for kernel selection. As shown in Table 14, for both yield stress and viscosity coefficient predictions, the GPR model with the Rational Quadratic (RQ) kernel yielded predicted values closest to the measured values. Compared with the Matérn kernel, the RQ kernel-based model achieved lower MSE and MAE and higher R^2^. Although the MSE of the RQ kernel (14.611) is slightly higher than that of the RBF kernel (14.609), the RQ kernel outperforms the RBF kernel in terms of MAE (3.0795 vs. 3.0850) and *R*^2^ (0.9459 vs. 0.9457), with only a negligible difference in MSE (0.002).

For yield stress prediction, all three kernel functions yielded satisfactory performance, with *R*^2^ values exceeding 0.9. Although the GPR model was able to estimate the viscosity coefficient to some extent, its prediction performance was relatively limited. This is attributed to the narrow range of viscosity coefficient values in the original dataset (ranging from 0.02 to 0.26), which made it difficult for the predicted values to closely match the measured values. Nevertheless, despite the highest *R*^2^ being only 0.8397 and the maximum MSE being 0.0267, the GPR model still provides meaningful predictions for the viscosity coefficient.

**Figure 13:**
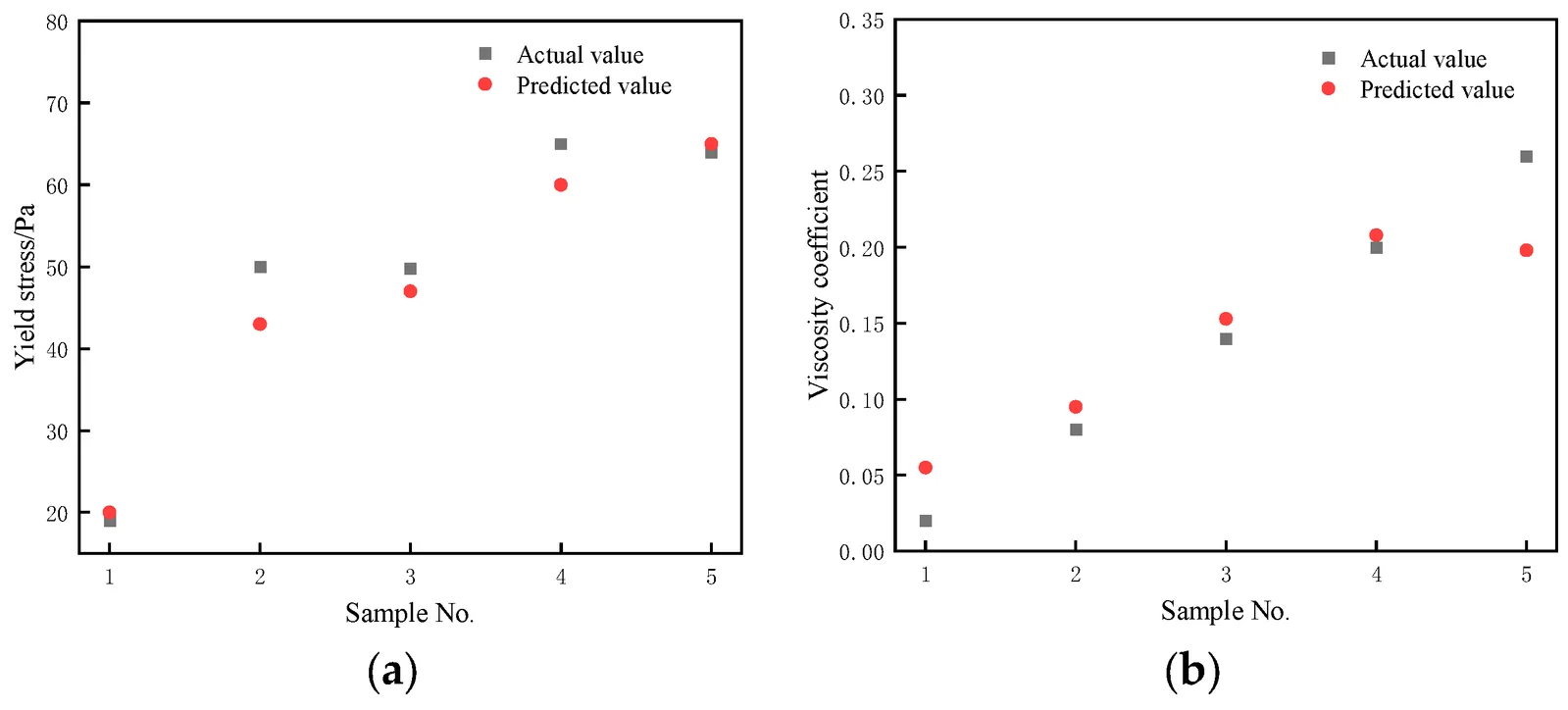
Comparison Plot of Predicted vs. Actual. (**a**) Yield stress; (**b**) Viscosity coefficient.

The comparison between the predicted and actual values of yield stress and viscosity coefficient on the test set using the RQ kernel-based GPR model is presented in Figure 13a,b, respectively. As shown in Figure 13a,b, the predicted results show reasonable agreement with the test set data for yield stress, while the viscosity coefficient predictions exhibit greater scatter, indicating generally acceptable predictions within the scope of the available dataset. This provides a potential new approach for studying the rheological parameters of unclassified tailings backfill slurry.

## 5. Optimization of Roof-Contact Backfilling Scheme for Large-Volume Stopes

**Figure 14:**
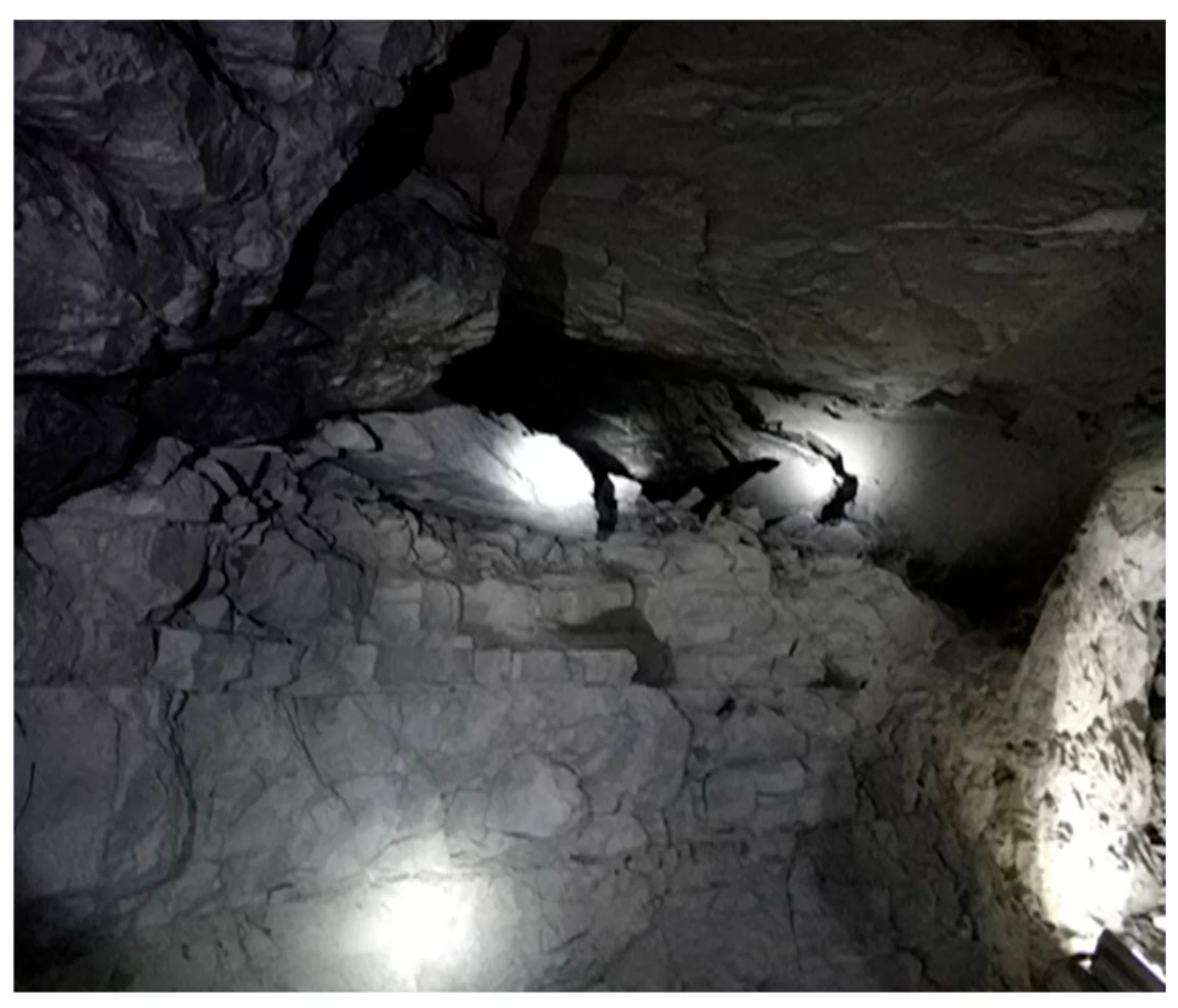
Schematic diagram of backfill body failing to contact the roof at the Daye Iron Mine.

The Daye Iron Mine employs an underground mining method, specifically the sublevel open stoping with subsequent backfill method, with the extraction sequence following an alternating pattern of mining one stope while leaving the adjacent one unmined. In the −180 m to −270 m level, the backfill body in the ore rooms has failed to achieve full roof contact due to the increased sublevel height (from 60 m to 90 m) and greater mining depth. Field investigations indicate that the average roof-contact rate in the ore rooms of the eastern district is only 63.33%, while that in the western district is even lower, at less than 60%. A schematic diagram of the incomplete roof contact in the western district is shown in Figure 14.

**Figure 15:**
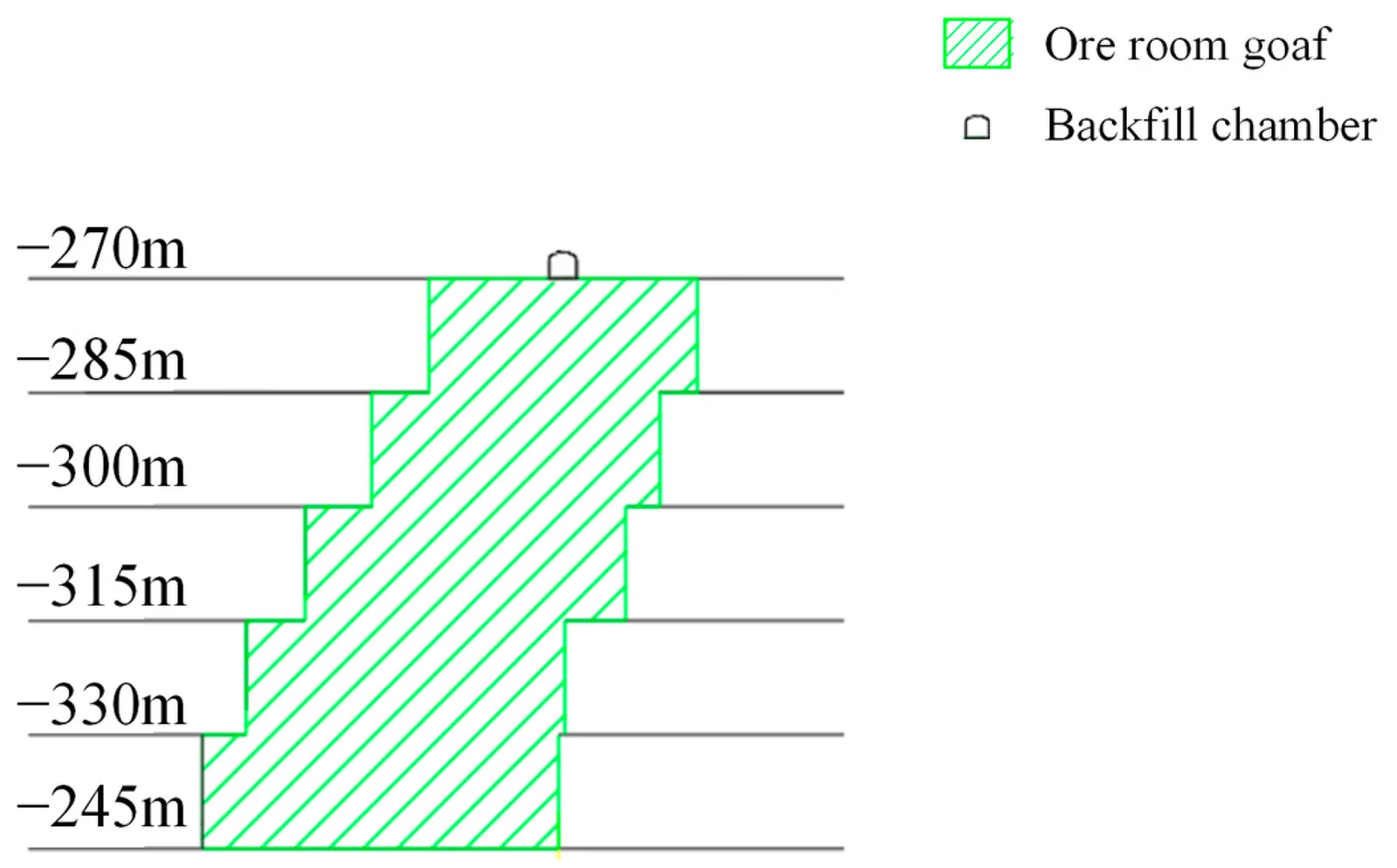
Schematic diagram of backfilling in Stope #3.

The Daye Iron Mine employs a cemented backfilling process for backfilling the mined-out voids. The current backfilling scheme adopts a single-point feeding configuration, with a filling chamber excavated on one side of the stope serving as the discharge point. Taking Stope #3 as an example, a schematic diagram of the backfilling arrangement is presented in Figure 15.

The cement-to-tailings ratio of the backfill slurry varies with the backfill location, 1:4 for ore rooms and 1:20 for pillars, with the original solids concentration being approximately 65%. Based on the spreadability and rheological test results of the present study, a slurry concentration of 71% can achieve gravity flow transport, while the flowability becomes extremely poor when the concentration exceeds 71%, and remains satisfactory when it is below 69%. However, according to previous studies on the shrinkage performance of backfill slurry [45], the shrinkage rate is relatively high when the concentration is below 69% and becomes minimal when the concentration exceeds 71%. Therefore, considering both the flowability and the shrinkage characteristics, the recommended slurry concentration for the Daye Iron Mine is recommended to be in the range of 69–71%.

However, at this concentration range, the yield stress and plastic viscosity of the slurry increase significantly, resulting in a pronounced self-flow accumulation angle in the stope, which adversely affects the roof-contact performance. To address this, the existing single-point feeding scheme was optimized using the Rocky-Fluent fluid-solid coupling numerical simulation method, based on the typical stope dimensions at the Daye Iron Mine. Six multi-point feeding schemes were designed for Stope #3 (length 40 m) and Stope #4 (length 90 m):

Case I: Single feeding pipe at the center of the 40 m stope;

Case II: Two feeding pipes with a spacing of 20 m for the 40 m stope;

Case III: Three feeding pipes with a spacing of 13.34 m for the 40 m stope;

Case IV: Three feeding pipes with a spacing of 30 m for the 90 m stope;

Case V: Four feeding pipes with a spacing of 22.5 m for the 90 m stope;

Case VI: Five feeding pipes with a spacing of 18 m for the 90 m stope.

**Figure 16:**
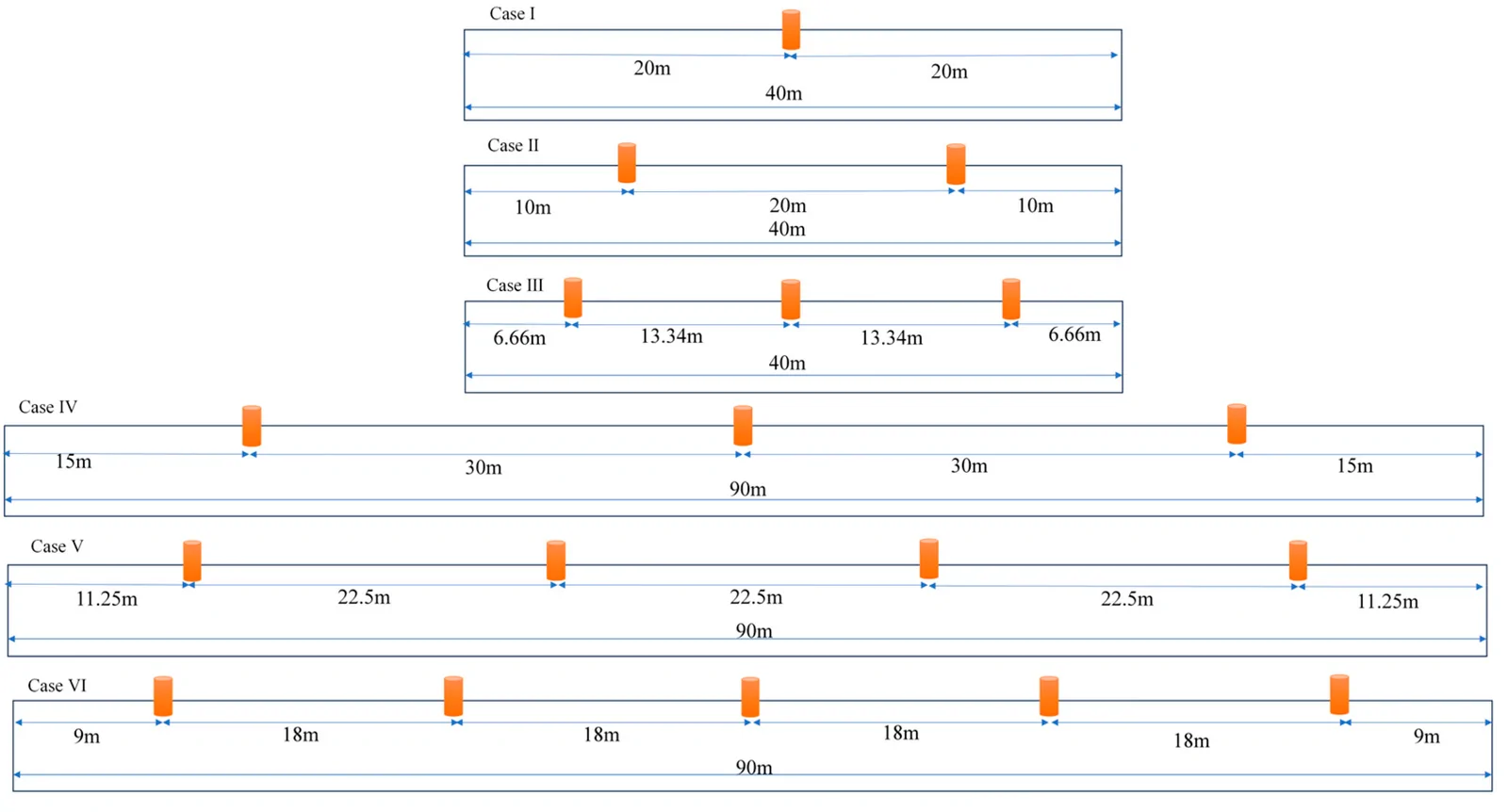
Optimized backfilling scheme.

The optimized backfilling schemes are illustrated in Figure 16.

**Figure 17:**
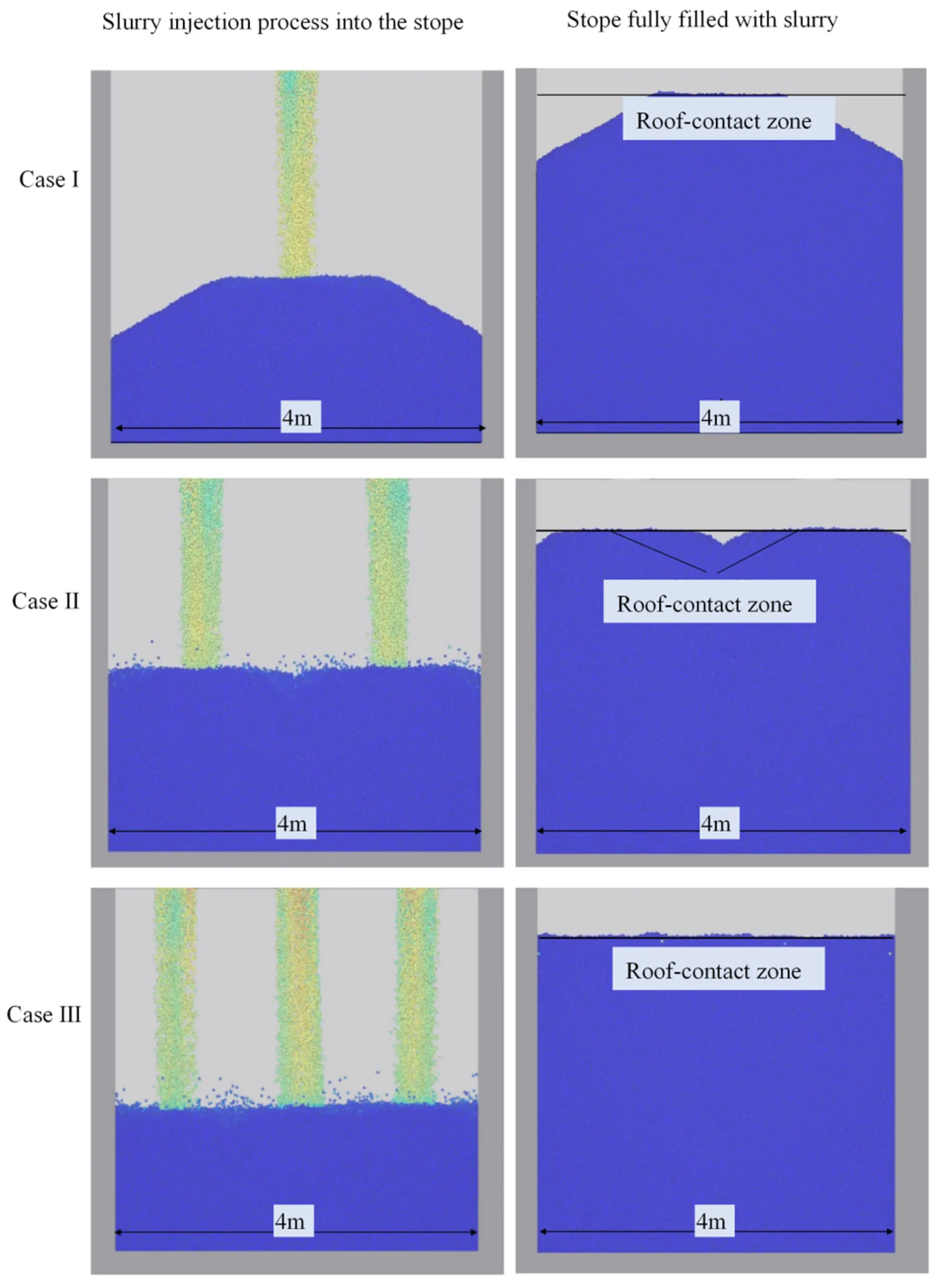
Numerical simulation result diagram for a stope length of 40 m.

**Figure 18:**
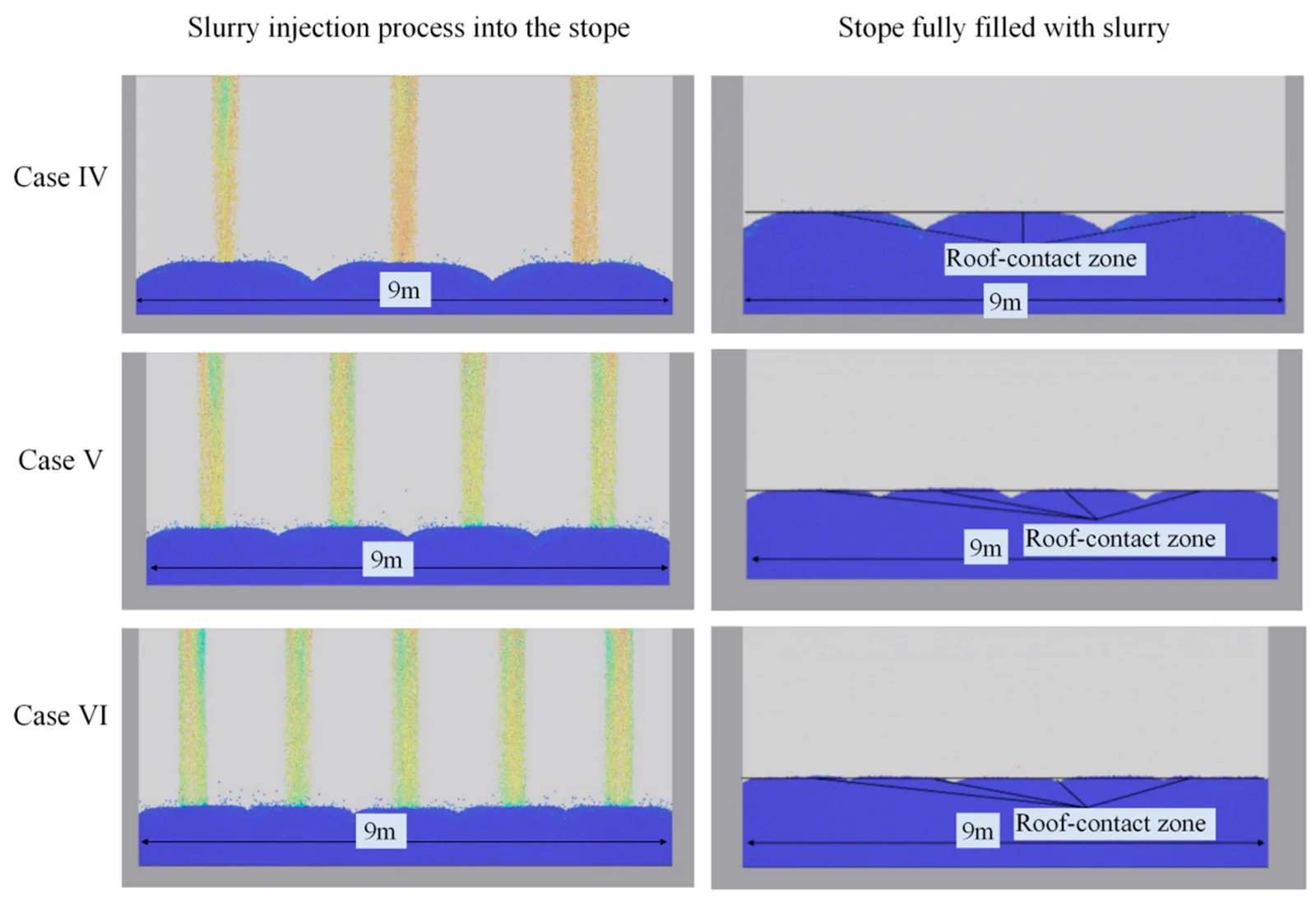
Numerical simulation result diagram for a stope length of 90 m.

Based on the rheological characteristics and parameters of the backfill slurry, three-dimensional stope models were established using Rocky (2025 R1)-Fluent (2025 R1) software, and numerical simulations were performed for the six schemes. The stope models were created at a 10:1 scale based on the actual stopes of the Daye Iron Mine. For the 40 m stope, the model dimensions are 4.0 m (length) × 1.5 m (width) × 4.0 m (height); for the 90 m stope, the model dimensions are 9.0 m × 1.5 m × 4.0 m. The mesh was generated using ANSYS (19.0) Meshing, with 33,731 nodes and 173,975 elements. The feeding pipe diameter at the slurry inlet is 0.5 m. The boundary conditions were set as velocity inlet (1.5 m/s) and pressure outlet. The Eulerian multiphase model with the k-epsilon turbulence model was employed, with water, air, and solid particles as the three phases. The solid particles were modeled as spheres with a diameter of 20 mm and a density of 2855 kg/m^3^. The inter-particle parameters were calibrated using the Box–Behnken experimental design, yielding a static friction coefficient of 0.8, a dynamic friction coefficient of 0.125, and a restitution coefficient of 0.238. A two-way Fluent-Rocky coupling approach was adopted, with Fluent solving the fluid phase (CPU-based) and Rocky solving the particle phase (GPU-based), and the Schiller–Naumann drag model was used. The simulation results are presented in Figure 17 and Figure 18.

The simulation results were analyzed, and the roof-contact rate was calculated as the ratio of the projected horizontal area covered by the solid particles to the total floor area of the stope model. The roof-contact ratios of the six backfilling schemes are summarized in Table 15.

As shown in Table 15, for the 40 m stope evaluated in this study, the use of three feeding pipes with a spacing of 13.34 m resulted in a roof-contact rate approaching 100%; for the 90 m stope evaluated in this study, the use of five feeding pipes with a spacing of 18 m achieved a roof-contact rate of 90.27%. Based on the six simulated configurations for stope lengths of 40 m and 90 m, it is recommended that the spacing between feeding pipes be controlled within the range of 13.34–18 m.

## 6. Conclusions

This study investigated the physicochemical properties, spreadability, rheological parameters, and flow characteristics of unclassified tailings backfill slurry through laboratory experiments and numerical simulations. Based on the experimental data from the L-type flow tester, an inversion analysis of the rheological parameters was conducted. Finally, the existing backfilling scheme at the Daye Iron Mine was optimized. Within the scope of the laboratory tests and numerical simulations conducted in this study, the main conclusions are as follows:

1. The unclassified tailings used at the Daye Iron Mine have a median particle size of 12.1 μm, with the primary reactive components being CaO, Al_2_O_3_, and MgO. The density of the tailings, measured using the pycnometer method, was 2.855 g·cm^−3^.
2. The spreadability test results indicate that, at a constant cement-to-tailings ratio, the flowability of the backfill slurry decreases with increasing solids concentration, with the slurries at concentrations of 73% and 75% exhibiting extremely low flowability. The rheological curves of the backfill slurry at different concentrations, measured using a rheometer, can be divided into three stages. The third stage of the rheological curves approximates a straight line, and within this high-shear-rate regime, the Bingham model serves as a practical engineering approximation for the determination of yield stress and plastic viscosity. Both yield stress and viscosity increase exponentially with increasing concentration. It should be noted that the full-range rheological behavior exhibits pronounced nonlinearity in the initial stage, which may be more accurately described by an exponential-type model or the Herschel–Bulkley model in future studies.
3. Based on the rheological parameters obtained from the physical experiments, the flow characteristics of the unclassified tailings backfill slurry in the L-type flow tester were numerically simulated using COMSOL Multiphysics (6.4). The flow velocity of the slurry in the horizontal channel decreases with increasing concentration. The flow regime of the slurry in the horizontal channel can be divided into three zones: the outlet zone, the steady-state zone, and the front zone. This flow regime zoning provides a reference for understanding slurry transport behavior in confined channels and offers a basis for subsequent optimization of roof-contact backfilling schemes.
4. A rheological parameter inversion analysis method based on Gaussian process regression (GPR) was proposed and validated using COMSOL (6.4)-generated flow data. The model was trained using numerical simulation results, and hyperparameter optimization was performed by maximizing the log marginal likelihood. Based on the comparative analysis of MSE, MAE, and *R*^2^, the GPR model with the rational quadratic (RQ) kernel demonstrated the best overall performance among the tested kernels, providing a potential new approach for estimating rheological parameters from observable flow data. The method is currently at the feasibility-validation stage and requires further experimental validation with physical L-type flow tests.
5. Based on the rheological parameters and characteristics of the backfill slurry investigated under the present laboratory and numerical conditions, the recommended slurry concentration for the Daye Iron Mine is 69–71%. For the 40 m stope evaluated in this study, the use of three feeding pipes with a spacing of 13.34 m can achieve a roof-contact rate approaching 100%. For the 90 m stope evaluated in this study, the use of five feeding pipes with a spacing of 18 m can achieve a roof-contact rate of 90.27%. Based on the six simulated configurations for stope lengths of 40 m and 90 m, the recommended pipe spacing is 13.34–18 m.
6. A limitation of this study is that the COMSOL (6.4) numerical simulation, the GPR-based inversion model, and the Rocky-Fluent backfilling scheme have not yet been validated against physical experiments or field data. The COMSOL (6.4) simulations, whose input rheological parameters were derived from rheometer measurements, serve as a trend predictor and data generator for the GPR inversion framework. The GPR model was trained and validated using only COMSOL (6.4)-generated data and is currently intended as a methodological framework to demonstrate the feasibility of the inversion approach. The Rocky-Fluent models were established based on actual stope geometries and conditions at the Daye Iron Mine, ensuring that the simulation inputs are physically grounded and representative of field conditions. Field backfilling tests are planned for future work once stope conditions become available, at which point the simulation results will be validated and calibrated with measured data. Future work will also involve conducting physical L-type flow tests to validate and calibrate the numerical models and the GPR inversion model, thereby further substantiating the reliability of the proposed methodology.

## Figures and Tables

**Table 1:** Particle size distribution of unclassified tailings from the Daye Iron Mine.

Particle Size (μm)	<1	1~5	5~10	10~25	25~50	50~100	>100
Differential percentage (%)	0.40	21.30	21.30	32.95	14.37	6.71	2.97
Cumulative percentage (%)	0.40	21.70	43.00	75.95	90.32	97.03	100

**Table 2:** Density test results.

Test No.	*K*_1_ (kg)	*K*_2_ (kg)	*K*_3_ (kg)	*K*_4_ (kg)	*G_s_* (kg·m^−3^)
1	0.06747	0.08667	0.17963	0.16698	2925
2	0.06745	0.08566	0.17843	0.16690	2721
3	0.06745	0.08805	0.18053	0.16697	2920
Average					2855

**Table 3:** Density of backfill slurry at different solids concentrations.

**Slurry Concentration (%)**	65	67	69	71
**Slurry Density (t/m^3^)**	1.746	1.787	1.830	1.876
**Density of Solid Dry Materials (t/m^3^)**	2.915			

The first column represents the table headers and is formatted in bold.

**Table 4:** Slump flow test results of backfill slurry at different solids concentrations.

Cement-to-Tailings Ratio	Slurry Concentration (%)	Lateral Spread (cm)	Longitudinal Spread (cm)	Average (cm)
1:4	75	11	11	11.00
	73	13	15	14.00
	71	15	17	16.00
	69	22.5	21	21.75
	67	39.5	39	39.25
	65	51	50	50.50

**Table 5:** Mathematical expressions of rheological models.

Rheological Model	Constitutive Equation	Fluid Type
Bingham model	$\tau =\tau_{0}+\eta \left(\frac{du}{dy}\right)$	Bingham fluid
H-B model	$\tau =\tau_{0}+\eta {\left(\frac{du}{dy}\right)}^{n}$	Yield-pseudoplastic fluid (*n* < 1)
	$\tau =\tau_{0}+\eta {\left(\frac{du}{dy}\right)}^{n}$	Yield-dilatant fluid (*n* > 1)

*τ*—the shear stress, Pa; *η*—the plastic viscosity, Pa·s; *τ*_0_—the initial yield stress, Pa; *n*—the flow index (dimensionless); *du*/*dy*—the shear rate, s^−1^.

**Table 6:** Fitting results of backfill slurry at different solids concentrations.

Slurry Concentration (%)	Fitted Equation	*τ*_0_ (Pa)	*η* (Pa·s)	Fitted Model
65	τ=19.81+0.02γ	19.81	0.02	Bingham
67	τ=40.62+0.03γ	40.62	0.03	Bingham
69	τ=70.03+0.06γ	70.03	0.06	Bingham
71	τ=89.37+0.16γ	89.37	0.16	Bingham

**Table 7:** Numerical Simulation Parameters.

Slurry Concentration (%)	Yield Stress (Pa)	Viscosity (Pa·s)	Density (t·m^−3^)
65	19.81	0.02	1.746
67	40.62	0.03	1.787
69	70.03	0.06	1.830
71	89.37	0.16	1.876

**Table 8:** Orthogonal Experiment Simulation Plan.

Test	Yield Stress (Pa)	Viscosity (Pa·s)	Density (kg·m^−3^)
1	20	0.02	1700
2	20	0.08	1750
3	20	0.14	1800
4	20	0.20	1850
5	20	0.26	1900
6	35	0.02	1750
7	35	0.08	1800
8	35	0.14	1850
9	35	0.20	1900
10	35	0.26	1700
11	50	0.02	1800
12	50	0.08	1850
13	50	0.14	1900
14	50	0.20	1700
15	50	0.26	1750
16	65	0.02	1850
17	65	0.08	1900
18	65	0.14	1700
19	65	0.20	1750
20	65	0.26	1800
21	80	0.02	1900
22	80	0.08	1700
23	80	0.14	1750
24	80	0.20	1800
25	80	0.26	1850

**Table 9:** Numerical Simulation Monitoring Results.

Time (s)					Flow Velocity (m·s^−1^)					Dip Angle (°)		
*T*_1_	*T*_2_	*T*_3_	*T*_4_	*T*_5_	*V*_1_	*V*_2_	*V*_3_	*V*_4_	*V*_5_	*δ*_1_	*δ*_2_	*δ*_3_
0.140	0.218	0.294	0.370	0.446	0.696	0.693	0.702	0.706	0.690	11.69	13.64	14.36
0.142	0.220	0.298	0.374	0.450	0.635	0.626	0.638	0.640	0.629	11.81	14.53	14.64
0.142	0.220	0.300	0.376	0.454	0.590	0.579	0.575	0.572	0.583	12.80	14.60	15.21
0.142	0.222	0.300	0.380	0.458	0.550	0.538	0.536	0.532	0.529	9.92	15.77	13.36
0.144	0.222	0.302	0.382	0.460	0.515	0.507	0.500	0.493	0.492	11.16	15.55	14.26
0.146	0.224	0.302	0.380	0.456	0.543	0.555	0.552	0.547	0.543	9.46	14.62	14.79
0.148	0.226	0.304	0.382	0.460	0.507	0.513	0.515	0.511	0.503	10.71	15.2	14.81
0.148	0.228	0.306	0.384	0.462	0.484	0.478	0.479	0.481	0.472	10.92	14.6	14.7
0.148	0.228	0.306	0.386	0.464	0.461	0.456	0.454	0.447	0.445	9.78	15.03	14.84
0.150	0.232	0.314	0.394	0.476	0.408	0.398	0.392	0.391	0.383	11.31	15.66	15.63
0.152	0.230	0.308	0.384	0.464	0.449	0.461	0.462	0.464	0.449	9.46	15.32	15.55
0.152	0.232	0.310	0.388	0.466	0.432	0.435	0.436	0.435	0.426	11.2	14.97	15.01
0.152	0.232	0.312	0.390	0.470	0.416	0.417	0.413	0.412	0.404	11.62	14.93	14.87
0.156	0.238	0.318	0.400	0.482	0.370	0.366	0.363	0.360	0.353	10.68	15.47	16.1
0.156	0.238	0.32	0.402	0.484	0.361	0.356	0.350	0.349	0.343	11.05	15.00	15.76
0.156	0.236	0.314	0.394	0.472	0.393	0.402	0.400	0.396	0.389	8.82	14.74	15.92
0.156	0.238	0.316	0.396	0.474	0.383	0.385	0.382	0.380	0.376	10.24	14.68	14.45
0.160	0.242	0.324	0.406	0.486	0.344	0.352	0.341	0.340	0.337	12.17	15.08	15.67
0.160	0.242	0.324	0.406	0.490	0.337	0.344	0.334	0.334	0.328	10.46	14.74	16.71
0.160	0.244	0.326	0.408	0.490	0.332	0.334	0.327	0.328	0.323	12.51	15.36	16.12
0.160	0.242	0.320	0.400	0.48	0.353	0.371	0.363	0.361	0.354	10.95	14.55	16.22
0.166	0.250	0.330	0.410	0.492	0.318	0.338	0.330	0.331	0.326	8.75	14.35	16.58
0.166	0.250	0.330	0.412	0.494	0.314	0.331	0.325	0.324	0.320	10.70	14.81	16.41
0.166	0.250	0.332	0.414	0.496	0.309	0.326	0.317	0.317	0.314	12.18	14.96	16.73
0.164	0.250	0.332	0.414	0.498	0.308	0.321	0.312	0.313	0.308	12.89	15.18	16.75

**Table 10:** Numerical Simulation Monitoring Results.

Time (s)					Flow Velocity (m·s^−1^)					Dip Angle (°)			Density (g·cm^−3)^
*T*_1_	*T*_2_	*T*_3_	*T*_4_	*T*_5_	*V*_1_	*V*_2_	*V*_3_	*V*_4_	*V*_5_	*δ*_1_	*δ*_2_	*δ*_3_	
0.00	0.00	0.00	0.00	0.00	1.00	1.00	1.00	1.00	1.00	0.71	0.00	0.29	0.00
0.08	0.06	0.11	0.09	0.08	0.84	0.82	0.84	0.83	0.84	0.74	0.42	0.38	0.25
0.08	0.06	0.16	0.14	0.15	0.73	0.69	0.67	0.66	0.72	0.98	0.45	0.55	0.5
0.08	0.13	0.16	0.23	0.23	0.62	0.58	0.57	0.56	0.58	0.28	1.00	0.00	0.75
0.15	0.13	0.21	0.27	0.27	0.53	0.50	0.48	0.46	0.48	0.58	0.90	0.27	1.00
0.23	0.19	0.21	0.23	0.19	0.61	0.63	0.62	0.60	0.62	0.17	0.46	0.42	0.25
0.31	0.25	0.26	0.27	0.27	0.51	0.52	0.52	0.50	0.51	0.47	0.73	0.43	0.5
0.31	0.31	0.32	0.32	0.31	0.45	0.42	0.43	0.43	0.43	0.52	0.45	0.40	0.75
0.31	0.31	0.32	0.36	0.35	0.39	0.36	0.36	0.34	0.36	0.25	0.65	0.44	1.00
0.38	0.44	0.53	0.55	0.58	0.26	0.21	0.21	0.20	0.20	0.62	0.95	0.67	0.00
0.46	0.38	0.37	0.32	0.35	0.36	0.38	0.38	0.38	0.37	0.17	0.79	0.65	0.50
0.46	0.44	0.42	0.41	0.38	0.32	0.31	0.32	0.31	0.31	0.59	0.62	0.49	0.75
0.46	0.44	0.47	0.45	0.46	0.28	0.26	0.26	0.25	0.25	0.69	0.61	0.45	1.00
0.62	0.63	0.63	0.68	0.69	0.16	0.12	0.13	0.12	0.12	0.47	0.86	0.81	0.00
0.62	0.63	0.68	0.73	0.73	0.14	0.09	0.10	0.09	0.09	0.56	0.64	0.71	0.25
0.62	0.56	0.53	0.55	0.50	0.22	0.22	0.23	0.21	0.21	0.02	0.52	0.76	0.75
0.62	0.63	0.58	0.59	0.54	0.19	0.17	0.18	0.17	0.18	0.36	0.49	0.32	1.00
0.77	0.75	0.79	0.82	0.77	0.09	0.08	0.07	0.07	0.08	0.83	0.68	0.68	0.00
0.77	0.75	0.79	0.82	0.85	0.07	0.06	0.06	0.05	0.05	0.41	0.52	0.99	0.25
0.77	0.81	0.84	0.86	0.85	0.06	0.03	0.04	0.04	0.04	0.91	0.81	0.81	0.50
0.77	0.75	0.68	0.68	0.65	0.12	0.13	0.13	0.12	0.12	0.53	0.43	0.84	1.00
1.00	1.00	0.95	0.91	0.88	0.03	0.05	0.05	0.05	0.05	0.00	0.33	0.95	0.00
1.00	1.00	0.95	0.95	0.92	0.02	0.03	0.03	0.03	0.03	0.47	0.55	0.90	0.25
1.00	1.00	1.00	1.00	0.96	0.00	0.01	0.01	0.01	0.02	0.83	0.62	0.99	0.50
0.92	1.00	1.00	1.00	1.00	0.00	0.00	0.00	0.00	0.00	1.00	0.72	1.00	0.75

**Table 11:** Normalized dependent variables of flow-state parameters of backfill slurry.

Test	Yield Stress (Pa)	Viscosity (Pa·s)
1	0.00	0.00
2	0.00	0.25
3	0.00	0.50
4	0.00	0.75
5	0.00	1.00
6	0.25	0.00
7	0.25	0.25
8	0.25	0.50
9	0.25	0.75
10	0.25	1.00
11	0.50	0.00
12	0.50	0.25
13	0.50	0.50
14	0.50	0.75
15	0.50	1.00
16	0.75	0.00
17	0.75	0.25
18	0.75	0.50
19	0.75	0.75
20	0.75	1.00
21	1.00	0.00
22	1.00	0.25
23	1.00	0.50
24	1.00	0.75
25	1.00	1.00

**Table 12:** Cross-validation results of GPR models with different kernels.

Target	Kernel	*R* ^2^	MAE	MSE	Δ*R*^2^
Yield stress	RBF	0.804 ± 0.267	4.634 ± 1.413	34.936	0.193
	Matern	0.820 ± 0.235	4.585 ± 1.420	32.896	0.180
	RQ	0.804 ± 0.265	4.665 ± 1.418	35.269	0.193
Viscosity	RBF	0.168 ± 0.607	0.0575 ± 0.0124	0.00464	0.832
	Matern	0.134 ± 0.690	0.0586 ± 0.0129	0.00479	0.866
	RQ	0.167 ± 0.609	0.0575 ± 0.0124	0.00465	0.833

**Table 13:** Leave-one-out cross-validation (LOOCV) performance comparison.

Target	Kernel	*R* ^2^	MAE	MSE
Yield stress	RBF	0.960	3.202	18.16
	Matern	0.959	3.332	18.47
	RQ	0.960	3.204	18.16
Viscosity	RBF	0.469	0.0511	0.00382
	Matern	0.465	0.0520	0.00385
	RQ	0.470	0.0511	0.00382

**Table 14:** Performance evaluation of rheological parameter prediction models under different kernel functions.

Kernel Function	Yield Stress			Viscosity		
	MAE	MSE	*R* ^2^	MAE	MSE	*R* ^2^
RBF	3.0850	14.609	0.9457	0.0355	0.0017	0.7639
Matern	4.3212	26.804	0.9007	0.0319	0.0014	0.8110
RQ	3.0795	14.611	0.9459	0.0267	0.0012	0.8397

**Table 15:** Roof-contact rates of different backfilling schemes.

Case	Stope Length (m)	Roof-Contact Rate (%)
I	40	35.25
II	40	49.89
III	40	100
IV	90	57.63
V	90	83.73
VI	90	90.27

## Data Availability

The original contributions presented in this study are included in the article. Further inquiries can be directed to the corresponding author.
